# Quantifying Soil Microbiome Abundance by Metatranscriptomics and Complementary Molecular Techniques—Cross‐Validation and Perspectives

**DOI:** 10.1111/1755-0998.14130

**Published:** 2025-06-03

**Authors:** Mathilde Borg Dahl, Stella Brachmann, Andrea Söllinger, Marina Schnell, Laureen Ahlers, Magdalena Wutkowska, Katharina J. Hoff, Neetika Nath, Verena Groß, Haitao Wang, Micha Weil, Marc Piecha, Marc Schaffer, Corinna Jensen, Andreas W. Kuss, Christoph Gall, Erika Wimmer, Thomas Pribasnig, Alexander Tøsdal Tveit, Bjarni D. Sigurdsson, Christa Schleper, Andreas Richter, Tim Urich

**Affiliations:** ^1^ Department of Bacterial Physiology University Greifswald Greifswald Germany; ^2^ Department of Arctic and Marine Biology UiT The Arctic University of Norway Tromsø Norway; ^3^ Institute of Soil Biology and Biogeochemistry, Biology Centre CAS České Budějovice Czech Republic; ^4^ Institute of Mathematics and Computer Science University Greifswald Greifswald Germany; ^5^ Institute of Bioinformatics University Medicine Greifswald Greifswald Germany; ^6^ Interfaculty Institute of Genetics and Functional Genomics University Greifswald Greifswald Germany; ^7^ Department of Microbiology and Ecosystem Science University Vienna Vienna Austria; ^8^ Archaea Biology and Ecogenomics Unit, Department of Functional and Evolutionary Ecology University of Vienna Vienna Austria; ^9^ Agricultural University of Iceland Hvanneyri Iceland

**Keywords:** biomass estimates, extraction standard, metatranscriptomics, quantitative transcriptomics, RNA

## Abstract

Linking meta‐omics and biogeochemistry approaches in soils has remained challenging. This study evaluates the use of an internal RNA extraction standard and its potential for making quantitative estimates of a given microbial community size (biomass) in soil metatranscriptomics. We evaluate commonly used laboratory protocols for RNA processing, metatranscriptomic sequencing and quantitative reverse transcription polymerase chain reaction (qRT‐PCR). Metatranscriptomic profiles from soil samples were generated using two library preparation protocols and prepared in triplicates. RNA extracted from pure cultures of *Saccharolobus solfataricus* was added to the samples as an internal nucleic acid extraction standard (NAE_std_). RNA reads originating from NAE_std_ were identified with a 99.9% accuracy. A remarkable replication consistency between triplicates was seen (average Bray–Curtis dissimilarity 0.03 ± 0.02), in addition to a clear library preparation bias. Nevertheless, the between‐sample pattern was not affected by library type. Estimates of 16S rRNA transcript abundance derived from qRT‐PCR experiments, NAE_std_ and a previously published quantification method of metatranscriptomics (hereafter qMeTra) were compared with microbial biomass carbon (MBC) and nitrogen (MBN) extracts. The derived biomass estimates differed by orders of magnitude. While most estimates were significantly correlated with each other, no correlation was observed between NAE_std_ and MBC extracts. We discuss how simultaneous changes in community size and the soils nucleic acid retention strength might hamper accurate biomass estimation. Adding NAE_std_ has the potential to shed important light on nucleic acid retention in the substance matrix (e.g., soil) during extraction.

## 1 Introduction

Modelling and predicting the fate of soil carbon (C) in a changing world has been a key scientific challenge for the past decades. Soil organic carbon (SOC) is released to the atmosphere as CO_2_ by mineralization, a process almost exclusively controlled by the soil (micro‐)biota. Temperate and cold regions, where microbial processes are often limited by temperature (Conant et al. 2011), hold the majority of Earth's soil carbon (Crowther et al. 2016). Therefore, especially in the light of climate change, there is a pressing uncertainty regarding the question as to how much C will be released to the atmosphere as a result of global warming?

The answer to that question lies in understanding SOC mineralisation. This, however, is not carried out by a single species but is rather a result of myriads of metabolic pathways of an unmatched diversity of microorganisms, that is the soil microbiome (Kramer et al. 2016; Nielsen et al. 2011). Understanding the pathways by which SOC is decomposed thus requires a comprehensive assessment of all relevant players in the soil (micro‐)biota community. Such assessment is hampered by the lack of visibility, difficulty in extractability, spatial heterogeneity, enormous diversity and temporal variation of soil (micro)biomes so that it is *de facto* still not possible (Anthony et al. 2023; Bonato Asato et al. 2023; Naylor et al. 2020). However, recent decades of methodological progress in the field of ‘molecular ecology’ (tightly linked to improved computational power and advances in the field of ‘bioinformatics’) have brought scientists closer to tackling the complexity of soil communities. A prominent example of such technique—which is nowadays widely used by soil ecologists—is environmental amplicon sequencing, a DNA‐based technique that enables the study of the relative abundance of target groups (Thompson et al. 2017). However, it does not allow for a comparison of absolute abundance (Alteio et al. 2021) and falls short in capturing microorganisms from different domains due to the lack of truly universal primers (Adl et al. 2014). Metatranscriptomics can overcome some of these limitations by being a primer‐independent approach, and by relying on the more quickly degraded RNA molecules instead of DNA, which can persist in the environment for a prolonged period after the death of an organism (Peng et al. 2024). Metatranscriptomics thus provide a cross‐domain census of potentially *active* bacteria, archaea and eukaryotes (i.e., through their ribosomes, rRNA), including multicellular organisms of higher trophic levels, as well as their transcriptional activity, through the characterisation of mRNA molecules (Urich et al. 2008). The metatranscriptomic approach has brought novel insights into soil microbiomes (Dahl et al. 2023; Schostag et al. 2019; Tveit et al. 2015). For example, the discovery that hitherto overlooked protist groups were highly abundant in a variety of soils (Geisen et al. 2015), and likewise, that predatory bacteria were highly abundant among micro‐predators in some soil types (Petters et al. 2021). In addition to that, metatranscriptomics also captures transcripts of viruses (in the non‐rRNA fraction), which have only recently been gaining attention for their role in soil nutrient cycling (Coclet et al. 2023; Starr et al. 2019). However, the extracted and sequenced RNA will mostly be ribosomal RNA (rRNA). This is why ribosomal depletion or mRNA enrichment are often applied if the aim is a functional analysis (mRNA); this, however, can impair the relative composition of the detected taxa in the sample (Gann et al. 2021; Tveit et al. 2014).

In the wake of lower costs for high‐throughput sequencing, the study of dynamic systems (requiring larger sample numbers) has become feasible (Täumer et al. 2022; Wang et al. 2023). Moreover, greater numbers of samples and deeper sequencing coverage may facilitate the identification of general biological patterns, which have recently provided new fundamental insights into microbial responses to a warming environment (Peng et al. 2018; Söllinger et al. 2022). However, this information‐rich data have yet to be translated into relevant parameters that could be integrated into large system‐scale models for SOC mineralization. A major obstacle in this context is the lack of a quantitative assessment of the organisms (Bradford et al. 2016; Filser et al. 2016). Quantitative soil metatranscriptomics, where the amount of total extracted RNA per sample is taken into account (Söllinger et al. 2018), has the potential to be a key technology in linking microbiome abundance and activity to biogeochemical processes of SOC mineralization mediated by the microbiome, as recently seen for methane gas flux measurements by Täumer et al. (2022). However, a known problem is that the amount of RNA, which can be extracted from a soil sample, will depend on the soil's chemistry, especially the adsorption strength of the soil, which increased with increasing clay and SOM content (Poursalavati et al. 2023; Paulin et al. 2013; Novinscak and Filion 2011).

Here, we employed a range of techniques to derive quantitative estimates of microbial biomass from molecular (RNA) data. We (i) applied an internal nucleic acid extraction standard (NAE_std_) for metatranscriptomics, (ii) evaluated the effect of library preparation for two common metatranscriptomic protocols on the resulting community profiles and standard recovery, (iii) performed quantitative reverse transcription polymerase chain reaction (qRT‐PCR) for broad range 16S and 18S rRNA quantification and finally, (iv) evaluated the agreement between the quantitative estimates derived from these molecular techniques and compared our results with other quantitative measures of soil microbial biomass (microbial biomass carbon, MBC and nitrogen, MBN).

## 2 Methods

### 2.1 Metatranscriptomics From Soil Samples

In this study, all samples originate from natural grassland sites in Iceland, which are part of the ‘ForHot’ experiment (www.forhot.is; Sigurdsson et al. 2016). The two grassland sites consist of replicated soil temperature gradients formed by natural geothermal warming of the bedrock below the soil profile. One site has experienced warming for more than 60 years and the second site developed similar temperature gradients after an earthquake in 2008, denoted long‐term warming (LTW) and medium‐term warming (MTW) transects, respectively. Samples were collected from four replicated plots with elevated (E) temperatures (+6°C and +9°C above ambient, LTW‐E_T_ and MTW‐E_T_, respectively) and corresponding samples from four replicated plots with ambient (A) temperatures (LTW‐A_T_ and MTW‐A_T_). Soil cores (0–10 cm layer; approx. 40 g) were collected from each plot at each site and immediately frozen on dry ice or in liquid nitrogen.

Since the origin of the samples is not of importance for this study, sample names have been simplified to sample type ‘A’ and ‘B’ (MTW and LTW, respectively), and number ‘one’ and ‘two’ (A and E, respectively).

**Dataset 1**: Soil samples collected from a single plot of type ‘A’ and ‘B’, ‘one’ and ‘two’ in October 2020 (*n* = 4). All samples were spiked with the internal Nucleic Acid Extraction standard (NAE_std_, see below) and processed in three technical replicates and prepared for sequencing with two different library protocols, thus representing 12 individual extractions (2× sites, 2× temperature conditions, 3× replicates) and *n* = 24 samples to be sequenced (12× extractions, 2× library preparation protocols). Detailed preparation protocols are described below.

**Dataset 2**: Soil samples collected from four replicated plots of type ‘B one’ and ‘B two’ in August 2021, October 2021, February 2022, May 2022 and July 2022 (*n* = 48). All samples were spiked with NAE_std_ and prepared for sequencing following the ‘nonAMP’ protocol described below.

**Dataset 3**: Previously obtained metatranscriptomic datasets of soil samples collected from four replicated plots of type ‘A’ and ‘B’, ‘one’ and ‘two’ in July 2016 (*n* = 16, Söllinger et al. 2022, Dahl et al. 2023). No standard was added to this data and sequence protocol is available form Dahl et al. (2023) and corresponds largely to the ‘AMP’ protocol described below, although with a higher sequencing depth of ca. 100 million reads per sample, using paired‐end sequencing (2x 125 bp) on a HighSeq2500 sequencer (Illumina, San Diego, USA).

### 2.2 Nucleic Acid Extraction Standard (NAE_std_ )—*Saccharolobus solfataricus*

In this study, an internal standard was added to all libraries by spiking all samples with 30 ng of RNA extract of high quality (RIN = 8.7, *R*NA *I*ntegrity *N*umber, a unite‐less scale from 1 to 10 obtained from 2100 Bioanalyzer; Agilent Technologies Inc. Santa Clara, CA, USA) from pure cultures of the archaeon *S. solfataricus* .

*S. solfataricus* is an aerobic sulfur‐oxidising thermoacidophile that grows optimally at 80°C and pH 3 (Zillig et al. 1980). Despite Iceland hosting the natural habitats of *S. solfataricus,* it is not expected to be able to live in the analysed soils and its RNA was never recovered in previous metatranscriptomic profiles from the sites (Söllinger et al. 2022; Dahl et al. 2023).

The cultures used for this study were part of an experiment where the virus SSV1 (*Taleaviricota*) of *Sulfolobus shibatae* , with introduced plasmid pUC18 for propagation in *Escherichia coli* (*Proteobacteria*) and the genes pyrEF of *S. solfataricus* as a selectable marker to complement pyrimidine auxotrophic mutants, had been introduced (Wimmer et al. 2024), and thus, traces of both the virus and *E. coli* were present in the dataset (see Section 3).

For detailed growth and extraction protocols, see Data S1.

**Dataset 4**: Four transcriptomic datasets from the same *S. solfataricus* pure cultures as those extracted and used for NAE_std_. The transcriptomic profiles consist of ca. 13 million reads per dataset, subjected to rRNA depletion to about 50% and paired‐end sequenced (2 × 150 bp). Data are deposited in NCBI (BioProject: PRJNA1099624).

Main features of the four datasets are summarised in Table 1.

**TABLE 1:** Summary of the main features of the four datasets used in this article.

	Dataset 1	Dataset 2	Dataset 3	Dataset 4
Time of sampling	Oct'20	Aug'21, Oct'21, Feb'22, May'22, Jul'22	Jul'16	2021
No. of samples	4 (×3 replicates)	48	16	4
Spiked with NAE_std_	Yes	Yes	No	—
RNA amplification applied	Yes (*n* = 12) and no (*n* = 12)	No	Yes	—
rRNA depletion applied	No	No	No	Yes (50%)
Sequenced libraries	24	48	16	4
Sequencing depth	Ca. 20 mio.	Ca. 20 mio.	Ca. 100 mio.	Ca. 13 mio.
Used for *in silico* mock	No	No	Yes	Yes
Used for qRT‐PCR	Yes (16S and 18S, Figure 2)	Yes (16S, Figure 3)	No	No

### 2.3 Laboratory Processing of Soil Samples

#### 2.3.1 RNA Extraction

RNA was extracted from each soil sample of dataset 1 in three technical replicates (of ca. 0.5 g fresh weight soil) using the RNeasy PowerSoil Total RNA Kit (Qiagen, Venlo, the Netherlands). Extractions were done following the manufacturer's protocol, but with the following modifications: each soil sample was subjected to two cycles of bead‐beating (for lysing and homogenization) with FastPrep‐24 5G (MP Biomedicals, Irvine, CA, USA) to maximise the recovery of RNA per sample, combined directly after phase separation in a joint capture tube. Furthermore, we spiked each sample with 30 ng of purified RNA of NAE_std_. The spiking (pipetting and a brief reverting of the tube) was done immediately after the homogenization and prior to the first‐phase separation during NA extraction. This procedure of adding the NAE_std_ directly to the soil‐buffer‐slurry mixture aims at exposing the NAE_std_ to the same soil chemistry as the released RNA from the soil community.

RNA extract was cleaned using column purification with MEGAclear Transcription Clean‐Up Kit (Thermo Fisher Scientific, Waltham, MA, USA). Finally, the RNA extract (50 μL) was mixed with 1.25 μL RNasin (1 μM) and stored at −70°C for later use, or subjected to quantitative and qualitative analysis.

RNA concentration was determined with Qubit RNA BR Assay Kit (Thermo Fisher Scientific, Waltham, MA, USA). RNA quality was analysed by Bioanalyzer using the Agilent RNA 6000 Nano Kit (Agilent Technologies, Santa Clara, CA, USA).

#### 2.3.2 Metatranscriptome Library Preparation

Bias in taxa abundance as a result of library preparation protocols is a known problem (Shi et al. 2021); here, we tested the effect of two common protocols, and thus for each sample in dataset 1 two sequencing libraries were built; one with and one without the application of the MessageAmp II‐Bacteria Prokaryotic RNA Amplification Kit (Thermo Fisher Scientific, Waltham, MA, USA; hereafter referred to as ‘AMP’). A kit designed for RNA amplification but often used to increase the relative proportion of mRNA due its preferential amplification of mRNA from the total RNA of a sample—a technique often used in metatranscriptomics studies when an analysis of the functional gene expression is the focus. Libraries were prepared using NEBNext Ultra II RNA Library Prep Kit for Illumina (New England BioLabs, Ipswich, MA, USA) following the manufacturer's protocol. RNA input was 100–120 ng. RNA fragmentation times were determined empirically to obtain the target fragment length of 370 bp (250 bp insert size +120 bp for adaptor/primer) for paired‐end sequencing. The applicable times were 3 min for amplified (‘AMP’) and 11 min for non‐amplified (‘nonAMP’) samples. Size selection for the desired insert size of 250 bp was performed with HighPrep PCR beads (MagBio Genomics Inc., Gaithersburg, MD, USA). The obtained fragments were confirmed by Bioanalyzer. The samples were paired‐end sequenced using a NextSeq 550 System using the NextSeq 500/550 High Output Kit v2.5 (300 Cycles; Illumina, San Diego, CA, USA) at the sequence facility at Greifswald University. Dataset 2 was prepared following the ‘nonAMP’ protocol. Raw sequencing files are available from NCBI (BioProject: RJNA1099624).

### 2.4 Bioinformatic NGS‐Data Processing

Raw sequences of dataset 1 and 2 were submitted to the PhyloFLASH processing pipeline following default settings (Gruber‐Vodicka et al. 2020). In brief, the pipeline identifies SSU rRNA sequences by aligning sequences to a modified SILVA v.138 (NR99; Yilmaz et al. 2014) database file using the BBmap mapping algorithm (Bushnell 2015), and all matches with a minimum identity of 70% (default) are kept, and taxonomic affiliation is assigned by the Last Common Ancestor (LCA) approach. For *Bacteria* and *Archaea*, the LCA taxonomy obtained from PhyloFLASH was directly carried over to the data analysis, whereas all sequences assigned to *Eukaryota* were extracted and reclassified using CREST4 (Lanzén et al. 2012). In CREST4, the *blastn* algorithm is used against a similarly modified SILVA v.138 (NR99) database also including the highly curated protist database PR2 v.4.13 (Vaulot et al. 2022). CREST4 taxonomic assignments are also based on the LCA approach with sequences that score within 2% of the bit score of the best alignment, provided that the best bit score is above the minimum value of 155. All bioinformatics analyses were performed on the CAPUT server hosted by the Kaderali Laboratory at Greifswald University.

#### 2.4.1 Classification Evaluation and Mock Samples

To ensure all sequences originating from the added nucleic acid extraction standard (NAE_std_) could be correctly identified in the post‐processing pipeline, dataset 3 (reference ‘forhot’ dataset without spiking) and 4 (pure culture transcriptomes of *S. solfataricus* ) were submitted to the identical bioinformatic pipeline for SSU rRNA taxonomic classification, as well as mixed mock samples of the two; total RNA reads from all four datasets (4x ‘forhot’ and 4x *S. solfataricus* ) were randomly subsampled to 200k sequences per dataset (for R1 and R2), merged (800k; 4x 200k for each), combined (2x 800k) and submitted to the bioinformatic pipeline for SSU rRNA‐based taxonomic classification. The taxonomic assignment was checked for all sequences originating from *S. solfataricus* .

Similarly, the mRNA sequences were evaluated for correct removal of the *S. solfataricus* sequences. Dataset 3 and 4 were sorted into SSU rRNA, LSU rRNA and non‐rRNA (putative mRNA) transcripts using SortMeRNA v.2.1 (Kopylova et al. 2012). Non‐rRNA sequences were then randomly subsampled and mixed in proportions of 10%, 1% or 0.1% of *S. solfataricus* sequences. In total, 32 mock samples (8 × 3‐levels spiking, and 8× non‐spiked controls) containing each 5 million reads were processed. The sequences were aligned with *blastn* using best‐match (default e‐value 10) against two custom build databases: one built from the four pure culture transcriptomes and one from 13 publicly available *S. solfataricus* genomes and SSV1 virus from NCBI (Table S5).

### 2.5 Data Analysis and Statistics

All statistical analyses were conducted in R.4.3.1 (R Core Team 2024) using mainly the ‘vegan’ (Oksanen et al. 2021), ‘ggplot2’ (Wickham 2016) and ‘reshape2’ (Wickham 2007) packages. NMDS ordination: SSU rRNA data were summarised to family level (*n* = 1816, including 65.5% of the community abundance). Ordination was calculated for a matrix of Bray–Curtis dissimilarities in two dimensions. Ordination stress was calculated and found to be low (0.1). Significant community dissimilarities between ‘A’, ‘B’, ‘one’ and ‘two’ were identified using a PERMANOVA (*n* = 999) with the ‘adonis2()’ function (‘vegan’ R package). Linear correlation tests: were performed using cor.test(methods=‘pearson’). Similarly, analysis of variances (ANOVA) was performed using aov() and post hoc tests were performed using the Tukey's Honest Significant Difference (HSD) test with the HSD.test() function from the ‘agricolae’ package in R (de Mendiburu 2023). Conceptual Figure 4 was drawn using BioRender (licence number EM26OP4E8E, BIoRender.com).

#### 2.5.1 Quantitative Reverse Transcription Polymerase Chain Reaction (qRT‐PCR)

In addition to estimating the number of SSU rRNA transcripts from the extracted RNA, qRT‐PCR was carried out for the raw RNA extracts. This was also done to shed further light on the relative abundance of *Pro*‐ and *Eukaryota*.

For this, general 16S SSU rRNA primers for *Prokaryota* defined by the earth microbiome project (EMP; 515F/806R, Apprill et al. 2015; Parada et al. 2016) were used together with eukaryotic 18S SSU rRNA primers known for their highest coverage in the SILVA database while exclusive to any prokaryotes (1183F/1443R, Hadziavdic et al. 2014). SSU rRNA genes were used together with standards of the same markers generated from *E. coli* and *Saccharomyces cerevisiae,* respectively. The standards were prepared by integrating the marker gene into a plasmid construct (pGEM‐T Easy Vector Systems, Promega, Germany), the *E. coli* JM109 transformants with the respective plasmid were cultivated at 37°C and 160 rpm overnight in liquid LB medium containing 100 μg/mL Ampicillin. Plasmids were extracted by using the Zyppy Plasmid Miniprep Kit (Zymo Research, Europe GmbH, Freiburg, Germany). Plasmids were linearised with *Sac‐I* restriction enzymes and the rCutSmart Buffer (New England Biolabs, Ipswich, MA, USA), followed by a transcription of the insert into RNA using the T7 transcription initiation site for the RNA polymerase (part of the pGEM‐T Easy plasmid construct). The length of the transcribed product was analysed with Bioanalyzer and the concentration with Qubit, whereafter the copy numbers were calculated as 340 Da (g mol^−1^ single nucleotides, nt) × fragment length (nt) × the Avogadro constant (6.022140857 × 10^23^ unites mol^−1^) and diluted to a series of 1 × 10^2^ to 1 × 10^8^ transcripts μL^−1^ for the qRT‐PCR. The qRT‐PCR standard curve was only accepted if the *R* ^2^ value was between 0.98 and 1.0, and the efficiency was between 0.9 and 1.10; negative controls were included using nuclease‐free water. Standard and 1:100 diluted RNA samples were amplified using 1 μL template iTaq Universal SYBR Green One‐Step Kit by Bio‐Rad laboratories (Bio‐Rad Laboratories GmbH, Feldkirchen, Germany) in a 15 μL reaction volume. qRT‐PCR was performed with a qTOWER^3^ G, PCR thermal cycler (Analytik Jena GmbH & Co. KG, Jena, Germany). Initial reverse transcription was performed at 50°C for 10 min, followed by PCR amplification: 95°C for 3 min; 35 cycles of 95°C for 30 s, 55°C for 30 s, 60°C for 45 s. All samples were subjected to melting curve analysis 60°C–95°C, 15 s, ΔT 1°C.

### 2.6 Microbial Biomass—MBC and MBN

Dissolved Organic Carbon (DOC) and Total Dissolved Nitrogen (TDN) were analysed from KCl extracts. In brief, an aliquot of 2 g of fresh soil (except for samples ‘B one’ and ‘B two’ Summer 2022, where only use 1 g was available) were suspended in 15 mL 1 M KCl and placed on an orbital shaker for 30 min at 200 rpm. A second aliquot of 2 g of fresh soil was fumigated with chloroform (CHCl_3_) for 48 h in the dark, and then extracted as described before. Extracts were subsequently filtered through ash‐free filter paper (Satorius 392 filters, Göttingen, Germany) and frozen at −20°C until analysed for dissolved organic carbon or nitrogen on a TOC/TDN analyzer (Shimadzu TOC‐L, TNM‐L). Microbial biomass carbon (MBC) and nitrogen (MBN) were calculated as the differences between dissolved organic carbon (DOC) and total dissolved nitrogen (TDN) content in the fumigated and non‐fumigated extracts, respectively. Values of MBC and MBN were corrected for incomplete extraction following standard corrections of 0.40 for N (Jonasson et al. 1996) and 0.45 for C (Joergensen 1996). To reach microbial biomass estimates, we multiplied MBC with two, assuming carbon to represent 50% of the total dry weight biomass (Bratbak and Dundas 1984) and for MBN 14% (Neidhardt et al. 1990) was adapted.

## 3 Results and Discussion

In this study, we applied a nucleic acid extraction standard (NAE_std_) for metatranscriptomics to estimate total RNA content in soil samples. A principle that has previously been applied in DNA amplicon studies (Zhang et al. 2022; Piwosz et al. 2018; Tkacz et al. 2018). For RNA sequencing, there is to our knowledge, only one other working group who has applied an internal extraction standard, and although successful, the application has not made it into common ‘best‐practice’ metatranscriptomic protocols, perhaps because the application was based on a custom synthesised plasmid as mRNA standard (Gifford et al. 2011; Moran et al. 2012). Here, we show how high‐quality ‘total RNA’ from the pure culture of a quasi ‘reference organism’, which is alien to the samples of a given study, can be applied as a standard. In our case, we used the very well‐described hyperthermophilic archaeon *S. solfataricus* of the class *Thermoprotei* in the phylum of *Crenarchaeota*. We demonstrate how the amendment of the standard can be used to make estimations of the total community size in the post‐processing data evaluation, and thereby to further approach a quantitative understanding of the studied microbiome.

### 3.1 Identification of NAE_std_ From the Annotated Sequence Profiles

First, we evaluated the correct removal of sequences originating from the NAE_std_ in our post‐sequencing data processing. We submitted ‘dataset 3’ (8× non‐spiked ‘forhot’ 2016 metatranscriptomic datasets, see Section 2) and ‘dataset 4’ (4× transcriptomes of *S. solfataricus* pure cultures) as well as *in silico* mixed mock samples of the two (datasets 3 and 4) to our SSU and mRNA classification pipeline.

From *S. solfataricus* pure cultures, a total of 5.17 × 10^7^ sequences were processed, of which 8.3 × 10^6^ were SSU rRNA and 1.7 × 10^7^ were identified as potential mRNA sequences. Among the SSU rRNA 99.9% were assigned to *Archaea* and of these < 1‰ were classified to *other* then the class *Thermoprotei* to which *S. solfataricus* belongs (Table S1). Of the 0.1% assigned to ‘Other’ 99% were identified as *E. coli* (the vector used as carrier of a mutant gene in the original experiment, see Section 2).

Similarly, the *S. solfataricus* non‐rRNA sequences (putative mRNA and viruses) were assigned to *Crenarchaeota* (99.9%), and ca. 0.1% to the experimentally introduced virus *Taleaviricota* and 0.05‰ *Proteobacteria* (the vector, Table S2).

In comparison, no reads were assigned to the class *Thermoprotei* in the non‐spiked environmental profiles (dataset 3). Here, between 4000 and 30,000 SSU rRNA reads were classified as belonging to *Archaea* and of these > 99% were assigned to the class *Nitrososphaeria*. The natural community composition *did* contain a low abundance of *E. coli* (< 0.01‰ of the bacterial reads, Table S3).

In the *in silico* mock samples for the SSU classification, only 0.08‰ of the NAE_std_ sequences were assigned to something other than expected—that is, 14 reads were assigned to genera of the bacterial family *Enterobacteriaceae* besides *Escherichia‐Shigella* (to which *E. coli* belongs, Table S4). For the non‐rRNA, a correct recovery of ≥ 99.9% was seen for all tested spiking levels (i.e., 10%, 1% or 0.1% of *S. solfataricus* non‐rRNA sequences, Table S6).

Thus, we conclude that if, in the down‐stream evaluation of samples spiked with *S. solfataricus* RNA, all reads assigned to the archaeon class *Thermoprotei* and the bacterial family *Enterobacteriaceae* are removed, the resulting profiles suffer only a negligible loss of the natural community (0.01% and 0.27% for SSU and non‐rRNA, respectively) and/or contamination (0.01‰ and 0.2‰ for SSU and non‐rRNA, respectively) of NAE_std_ sequences.

### 3.2 The Effect of Library Preparation

**FIGURE 1:**
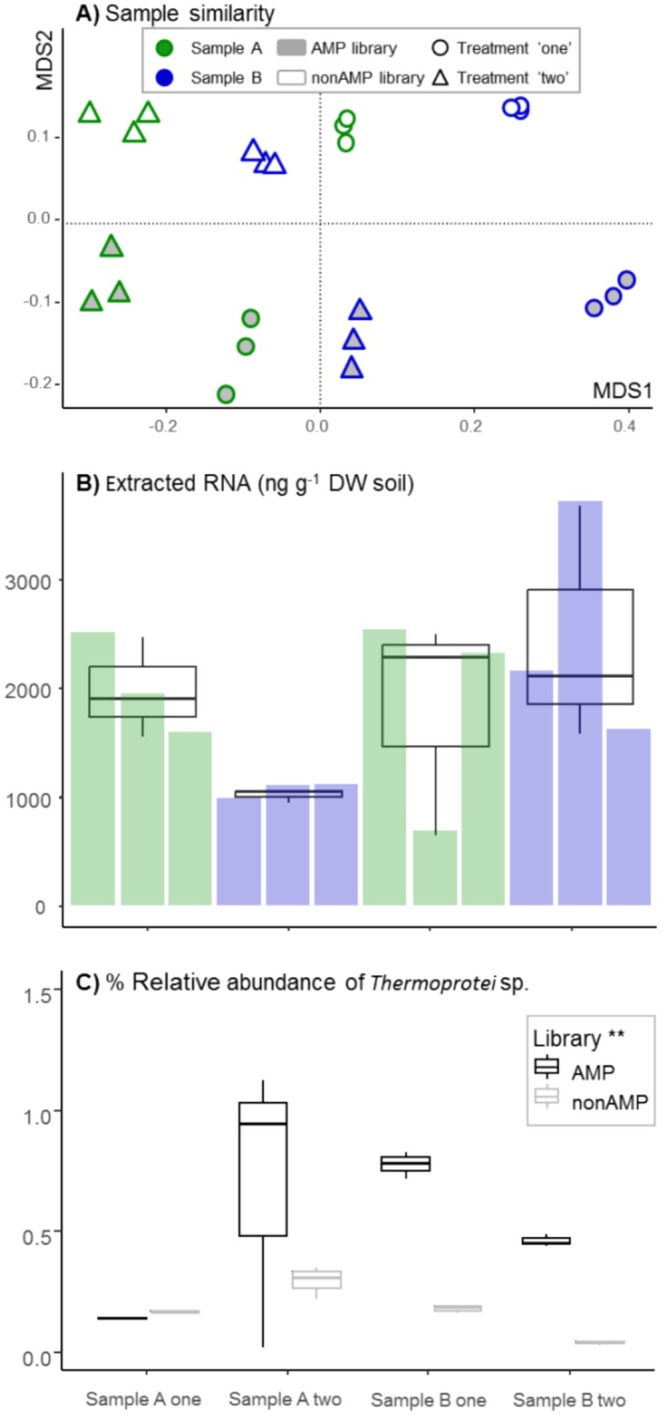
Similarity and replication consistency between samples obtained by two different library protocols. NMDS ordination of the community profiles obtained from taxonomic classification of rRNA, (A). The total extracted RNA g^−1^ DW soil, (B). The *per cent* relative abundance of SSU rRNA reads assigned to the order *Thermoprotei* or deeper. Boxplot showing 25% and 75% quantiles, median (horizontal line) and 5% and 95% quantiles (whiskers), (C). Significant differences tested with two‐way ANOVA, **p < 0.01 (*p* = 0.003).

A very high replicability was seen for the community profiles (i.e., tight clustering of the three libraries/extractions of the same sample from dataset 1, Figure 1A) with Bray–Curtis dissimilarity of 0.039 ± 0.024 and 0.027 ± 0.007 for ‘AMP’ and ‘nonAMP’ replicate samples, respectively. However, equally noteworthy was the differentiation between libraries with and without amplification treatments (i.e., ‘AMP’ and ‘nonAMP’ respectively; seen as a separation above and below zero on the 2nd NMDS axis in Figure 1A), stressing the well‐known but less studied problems associated with comparing community compositions across datasets obtained by different protocols (Gann et al. 2021; Tveit et al. 2014). Despite this clear methodological bias, the ‘ecological signal’ was preserved for both library types, separating ‘Sample’ and ‘Treatment’ along the 1st NMDS axis.

As expected, the relative abundance of NAE_std_ (sequences assigned to *Thermoprotei* sp.) was inversely correlated to the amount of extracted RNA (Figure 1B), where more RNA resulted in a lower abundance of the standard (i.e., a dilution effect), and a Pearson's correlation coefficient *r* = −0.6, *p* = 0.04, for nonAMP libraries was seen. This relationship was, however, non‐significant for AMP libraries (*r* = −0.2, *p* = 0.54, and when considering one outlier for Sample A‐two *r* = −0.4, *p* = 0.2). Although the between‐sample pattern was kept across library types, the relative abundance of NAE_std_ was significantly affected by the library protocols, where a shift of ca. +0.4% was seen for AMP libraries (*p* < 0.001, Figure 1C).

**FIGURE 2:**
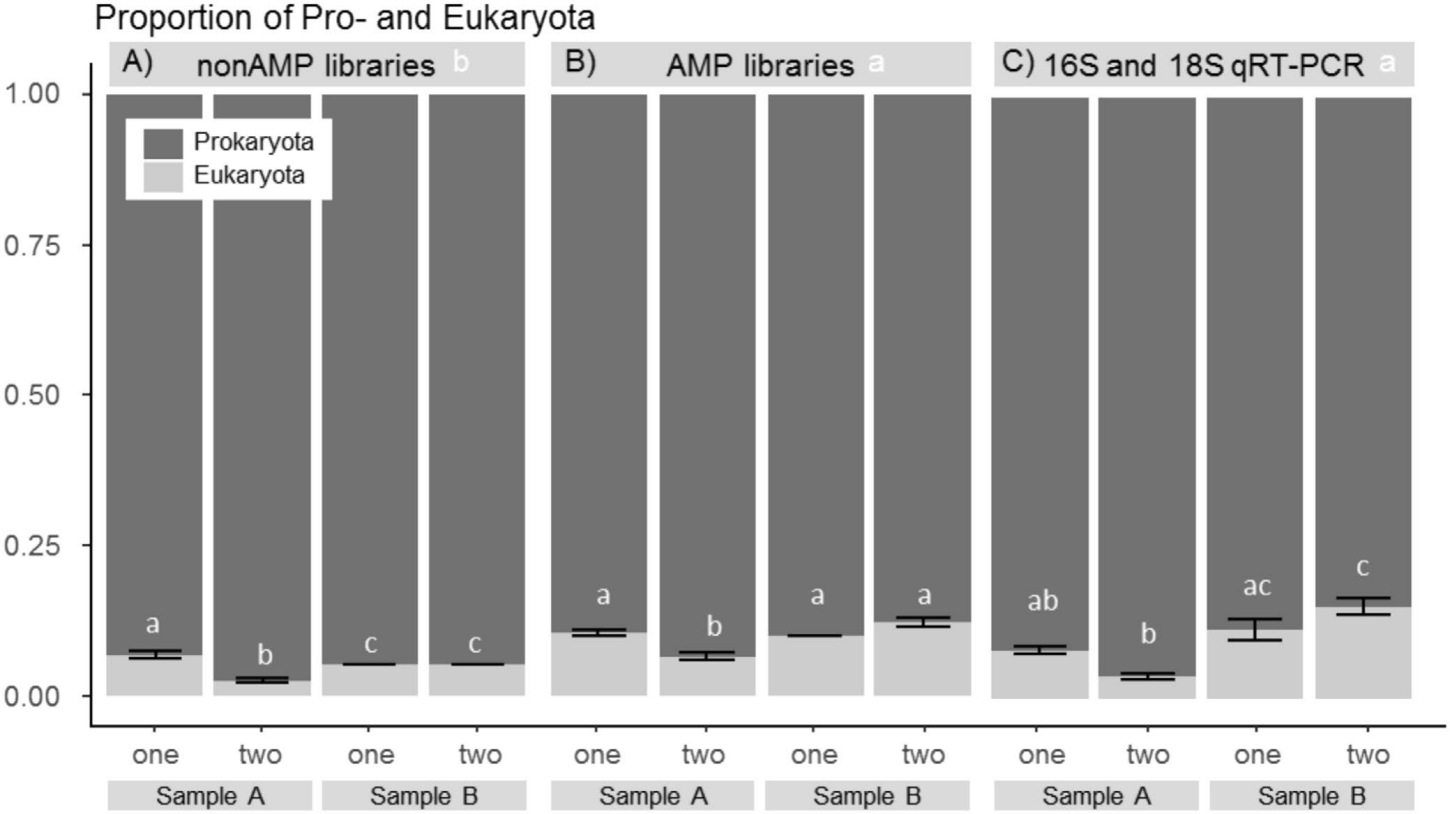
Average relative proportion of the *Pro*‐ and *Eukaryota* in the community per sample, shown for nonAMP (A) and AMP (B) sequenced libraries and qRT‐PCR reads of 16S and 18S rRNA (primer pair; 1183F/1443R (16S) and 515F/806R (18S); C). Significant differences were tested with two‐way ANOVA, followed by Tukey's HSD post hoc test for between‐sample statistics separately for each dataset A to C. Significant differences (*p* < 0.05) are indicated with letters a, b, c both between methods (header) and sample type (on bars).

In the annotated SSU rRNA community profiles, the domain‐level composition between samples was very similar for the two library types, with a significantly lower proportion of *Eukaryota* seen in Sample A‐two for both library types (Figure 2A,B) as well as the eukaryotic proportion being equal between Sample B‐one and B‐two, although significantly distinguishable from Sample A‐one only in nonAMP libraries.

However, a small but significant shift in the overall proportion of pro‐ and eukaryotic taxa for the two libraries was seen (*p* < 0.001, Figure 2A,B). On average, prokaryotes accounted for 90.2% ± 2.3% and 95.0% ± 1.7%, and eukaryotes for 9.8% ± 2.3% and 5.0% ± 1.7% in AMP and non‐AMP libraries, respectively. In an attempt to shed light on this relationship, qRT‐PCR was performed on the raw RNA extracts of the samples. In this method, RNA is reverse transcribed (RT) into DNA and targeted using general 16S and 18S rRNA primers (for details, see Section 2). It should be noted that the primers are, albeit general, not truly universal, and thus, we must consider the risk that some taxa are overlooked by either marker, compromising a true validation (Vaulot et al. 2022; Wasimuddin et al. 2020).

Similar to the observations for the sequenced profiles, also qRT‐PCR displayed a high replication consistency between each RNA extract of the same sample type, and when combining the number of transcripts obtained from the general 16S and 18S rRNA primers, the obtained proportions were very consistent and the between‐sample pattern strongly reflected the one observed in the sequenced profiles (Figure 2C). In absolute numbers, an average proportion of 91.5% and 8.5% for *Pro*‐ and *Eukaryota*, respectively was obtained with qRT‐PCR. These numbers best resembled—and were correlated to—the AMP library profiles (*r* = 0.74, *p* < 0.05), whereas no significant correlation was seen for nonAMP libraries (*r* = 0.43, *p* = 0.2).

Looking deeper into the sequenced profiles, we found that the increase of *Eukaryota* seemed not to be driven by specific taxa being positively biased in AMP libraries, but rather because all major eukaryotic groups had a slightly higher relative abundance (with significant differences only for higher plants, *Embryophyta*, and the protists *Hacrobia*, *Foraminifera* as well as the supergroup *Amorphea*, Data S3). Despite the differences highlighted here, we conclude a high overall congruency in the obtained profiles between both library protocols and qRT‐PCR.

### 3.3 Quantifying Communities From Sequences SSU rRNA Transcripts

In the following, we estimate the number of SSU rRNA transcripts per gram dry weight (DW) soil and assess the consistency of proxies by comparing the number of transcripts obtained from (i) the quantitative metatranscriptomic approach described in Söllinger et al. (2018), (ii) the abundance of our added nucleic acid extraction standard ( *S. solfataricus* described above) and (iii) 16S rRNA reads from qRT‐PCR. For simplicity, we only consider 16S rRNA, since > 90% of the annotated SSU of these samples were prokaryotes. We also adjusted the relative abundance of the *Prokaryota* (i.e., 16S rRNA) to reflect the weight difference between pro‐ and eukaryotic SSU rRNA, using an assumed average size of 1500 nt for 16S rRNA (Yilmaz et al. 2011) and 1700 nt for 18S rRNA (average 18S length obtained from the Silva database, 15. March 2024). Similar calculations can be conducted for 18S rRNA, but some of the conversion factors used below are harder to parameterise for *Eukaryota*, increasing the uncertainty for the obtained results. For this evaluation, dataset 2 was used, *n* = 46 (two samples were removed as outliers with 50% and 5% relative abundance of reads assigned to the internal standard, *Thermoprotei* sp., vs. an average of 0.9% ± 0.7% for the remaining dataset, see Data S4. These were removed in all following analyses).

- Quantitative metatranscriptomic approach

Söllinger et al. (2018) established quantitative transcriptomics (hereafter qMeTra) by integrating relative SSU rRNA read abundance with known molecular attributes (e.g., the molar mass of nucleotides) and the total extracted RNA per sample. By applying this method, it was demonstrated how the mass of transcripts (μg transcripts g^−1^ sample) in a time series (unlike the relative abundance) revealed a dynamic profile of the community size and transcriptional activity of methanogens in cow rumen (Söllinger et al. 2018) and soil (Täumer et al. 2022; Wang et al. 2023) matching gas flux data.

To estimate the SSU rRNA content of each sample following the qMeTra approach (Söllinger et al. 2018), the following assumptions and definitions were adopted:

- A theoretical ratio of 4:96 for mRNA:rRNA, following Neidhardt and Umbarger (1996) who determined mRNA to account for 4% of the total RNA by mass (in Moran et al. 2012), corresponds well to the empirically determined 3% ± 1% of non‐rRNA sequences in dataset 2 (Data S4).
- The length of prokaryotic SSU and LSU transcripts was considered to be ca. 1500 nt and 2900 nt, respectively (Yilmaz et al. 2011). Thus, we defined the SSU rRNA fraction as 1/3 of the rRNA (i.e., 1/3 of 96% ≈ 32% of the total RNA was considered to be SSU rRNA).

This resulted in an estimated 1235 ± 556 ng SSU rRNA g^−1^ dry weight (DW) soil. Hereafter follows to estimate how many 16S rRNA transcripts this represents. In line with Söllinger et al. (2018) we used this definition:

- One single 16S transcript has the average molar weight of 4.5 × 10^5^ Da (1500 × 320.5 Da + 159 Da accounting for 5′ triphosphate per nucleotide; 1 Da = 1 g mol^−1^).

In combination with the Avogadro constant of ca. 6.02 × 10^23^ units per mol, an estimated 1.22 × 10^9^ 16S rRNA transcripts per ng RNA is reached, resulting in an estimated 1.4 × 10^12^ ± 6.3 × 10^11^ 16S rRNA transcripts g^−1^ DW soil for dataset 2.

ii Quantitative NAE_std_ approach

The relative abundance of NAE_std_ in the SSU rRNA annotated data was 0.9% ± 0.7%. This is considerably lower than expected when purely considering the ratio between added (30 ng) and extracted RNA (839 ± 374 ng per ca. 0.5 g WW soil, Data S4) from which an expected average abundance would be ca. 3.6% (30 ng/839 ng). This discrepancy may be alternatively explained as follows:

- the added RNA was not fully homogeneously integrated into the suspension and/or retained at the same rate as the abundant RNA, both unlikely given the replication consistency (see dataset 1, Figure 1C).
- the added RNA degraded faster than the abundant RNA (we see no apparent reason for this).
- the obtained RNA does not reflect a complete RNA extraction, that is, some RNA remains bound to soil particles as a result of the specific chemistry for a given soil (as seen in Thorn et al. 2019), so that the assumption of a full recovery of 30 ng would lead to an overestimation of the NAE_std_ abundance. This seems the most likely explanation.

If the explanation in (iii) would be the only one, we could rely on the relative abundance obtained from the sequencing profiles to estimate the full RNA content of the sample. Thus, by knowing that the spiked‐in 30 ng RNA resulted in a relative abundance of 0.9% ± 0.7% we can simply scale back to estimate how many ng would correspond to a 100% extraction of the sample:

$$\begin{array}{c} \left(\frac{\text{spiked}-\text{in}\;\mathrm{RNA}\;{\left(\mathrm{ng}\right)}_{\mathrm{Sx}}}{\%{\mathrm{NAE}}_{\mathrm{std}}\;{\text{abundance}}_{\mathrm{Sx}}}\times 100\right)-\text{spiked in}\;\mathrm{RNA}\;{\left(\mathrm{ng}\right)}_{\mathrm{Sx}}\\ =\text{Total estimated}\;\mathrm{ng}\;\mathrm{RNA}\;{\text{in sample}}_{\mathrm{Sx}} \end{array}$$

By this, we arrive at an estimated average RNA content of 42.0 ± 48.1 μg g^−1^ DW soil. Noteworthy, we see here a very large standard variation of the numbers, which display a much larger range than the values obtained for the extracted RNA. If we consider the median value, this was 18.4 μg g^−1^ DW soil, which is ca. 5× higher than what was obtained during RNA extractions 3.9 ± 1.7 μg g^−1^ DW soil.

From here, the approach is similar to the qMeTra calculation above:

The quality of the RNA of both the NAE_std_ and environmental RNA was high (not degraded) as seen from the Bioanalyzer RNA integrity number (a unite‐less scale from 1 to 10; average RIN > 8, Data S5). Thus, we assumed that the theoretical composition of the RNA (as described above) was intact and applied the same assumption as stated above for the 16S rRNA content and weight, which resulted in an estimated 1.6 × 10^13^ ± 1.8 × 10^13^ 16S rRNA transcripts g^−1^ DW soil.

iii Quantitative PCR approach

As for dataset 1, the general EMP 16S rRNA primers were used in the qRT‐PCR assay for dataset 2. As mentioned above, the input RNA was of high quality (average RIN > 8, Data S5) and the SSU molecules were considered to a large extent to be full‐length.

qRT‐PCR assays with the 16S primers yielded an estimated 2.73 × 10^10^ ± 1.7 × 10^10^ 16S rRNA transcript g^−1^ DW soil.

#### 3.3.1 Comparison of Biomass Proxies

**FIGURE 3:**
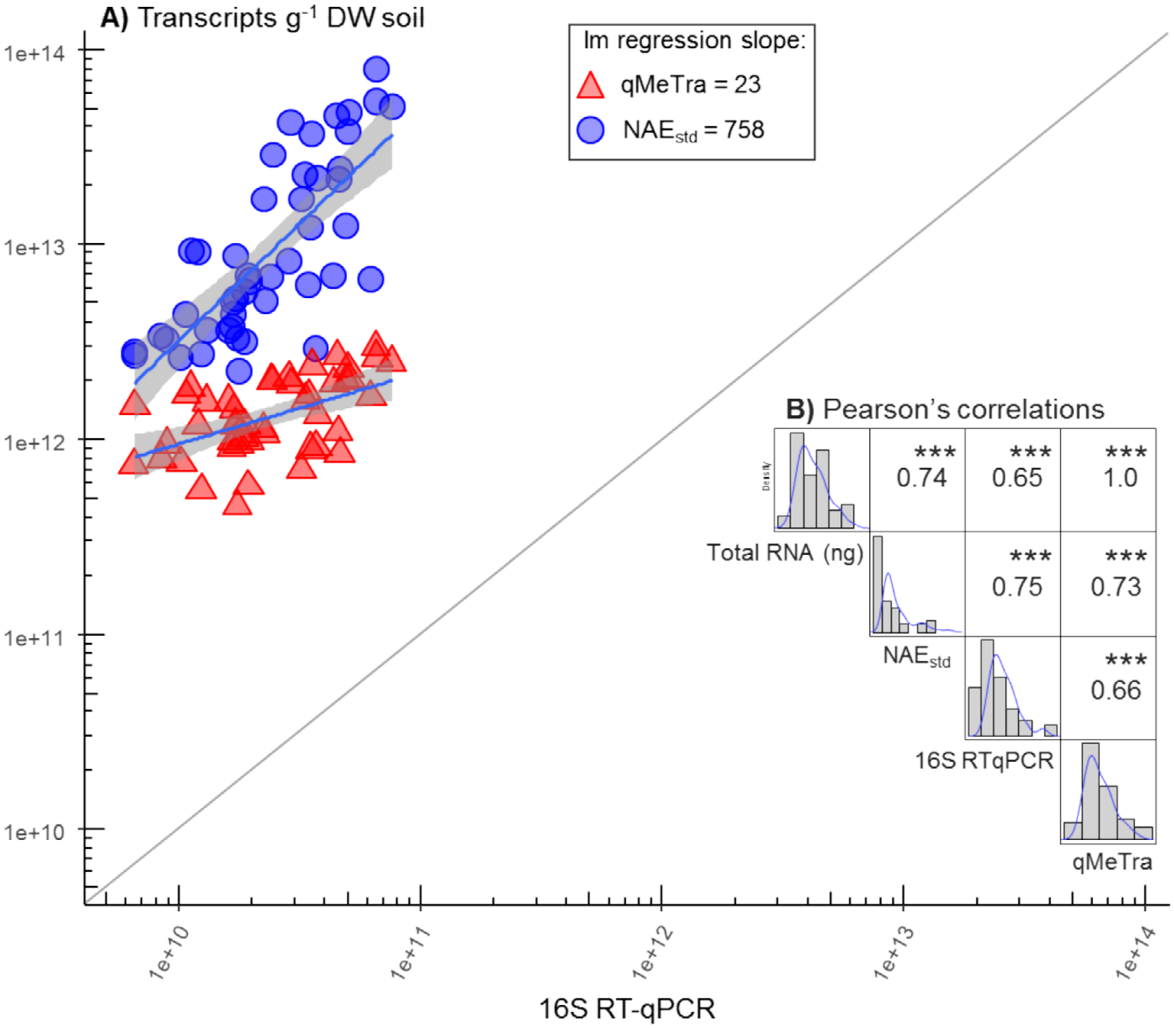
(A) log–log plot of the estimated number of 16S transcripts g^−1^ DW soil by NAE_std_ or qMeTra (*y*‐axis) and qRT‐PCR (*x*‐axis) and their linear regression (the slope is given in the legend). Diagonal line represents the 1:1 line. (B) Person's *r* correlation matrix for all estimates including the total extracted RNA from the samples. Significant correlations are indicated with ***p < 0.001 (*n* = 46).

Despite differing by orders of magnitude in absolute numbers, the three 16S rRNA transcript estimates derived from the methods presented above (qMeTra, NAE_std_ and qRT‐PCR quantifications) were strongly correlated (Pearson's *r* approaching 0.7 for all cross‐correlations, *p* < 0.001; Figure 3B). Likewise, the large range difference for each estimate was seen from the slopes of the linear regressions (Figure 3A).

Assuming all SSU rRNA in a given RNA extract was intact (and accessible for primers), a theoretical relationship between qMeTra and qRT‐PCR of 1:1 would be expected (i.e., diagonal line in Figure 3A), since they both relate directly to the RNA extract of each sample. Here, we found a ca. 1:53 relationship between qMeTra and qRT‐PCR mean values, suggesting that only ca. 2% of the SSU rRNA was detected in the qRT‐PCR reaction (which seems low, given the high RNA integrity, Data S5). Contrary to that, the NAE_std_ has the potential to reflect the non‐extractable part of the RNA content of a sample and thus is expected to result in higher SSU rRNA transcript estimates than the others for any non‐complete RNA extraction (in accordance with the presented results). However, the ca. 1:573 relationship found between mean values of NAE_std_ and qRT‐PCR, would suggests that only ca. 0.2% of the total RNA was extracted. This proportion appears at first glance to be unrealistically low. However, it is worth noting that Paulin et al. (2013) found, that only when adding 1000 μg g^−1^ soil of an ‘adsorption‐site competitor’ (to alleviate adsorption of RNA to soil particles) during RNA extraction of soil with a high clay content, the detection of their control mRNA marker was possible. The same experiment found an 80% DNA retention in the soil, but compared to DNA, RNA is expected to adsorb stronger than DNA to soil particles due to more hydroxyl groups (Cleaves et al. 2010). Moreover, the adsorption forces are expected to be particularly high in Andosols—soils of the volcanic origin used in this study, due to the presence of allophanes and imogolites (Wang et al. 2012) and high water retention (without causing anoxic conditions, Sigurdsson et al. 2016). Finally, adsorption as a function of time represents a potentially important aspect, which was not addressed in the current study (here all samples were processed in batches of less than six samples and the NAE_std_ was added ‘immediately’ upon lysis, as a reference time interval, Paulin et al. (2013) used a 5‐min incubation time for their ‘adsorption‐site competitor’).

With the overarching aim of quantification, the correlation between the presented estimated numbers of transcripts in relation to independent measures of microbial biomass will not provide a closer understanding of which method performs better as a proxy for the community biomass. Thus, we must attempt to bring the transcripts on a scale comparable to the available measures, in this case microbial biomass carbon (MBC) and nitrogen (MBN). To achieve this, we converted the estimated number of 16S rRNA transcripts to a potential number of prokaryotic cells and the biomass they represent. This was achieved by applying the following two extremely crude assumptions:

- All active (i.e., present in the datasets) prokaryotes contain 20,000 ribosomes per cell (Matamouros et al. 2023; Milo and Phillips 2015) and,
- The weight of one prokaryotic cell is 3 × 10^−13^ g DW biomass (Milo and Phillips 2015; Kim and Gadd 2008).

Both of these values are determined from *E. coli* under laboratory conditions. Although soil *bacteria* are generally considered to have a smaller cell sizes than those observed under laboratory conditions (Portillo et al. 2013), community members might be larger than *E. coli* . For example, Portillo et al. 2013 found *Proteobacteria* and *Actinobacteria* more abundant in larger size fractions (> 3 μm diameter); these phyla made up ca. 35% of the *bacteria* in the analysed samples. However, intrinsic (e.g., growth phase, Portillo et al. 2013) and environmental (e.g., temperature, Söllinger et al. 2024) factors aside, a linear relationship is expected to exist between cell size and ribosomal content (Matamouros et al. 2023), thus counterbalancing the calculation of biomass (i.e., increasing the assumed number of ribosomes per cells would result in a lower estimated cell number, but each cell would subsequently be assumed to weigh more). We ignore for now the eukaryotic proportion of the biomass.

The first conversion (20,000 ribosomes per prokaryotic cell) resulted in an estimated average number of prokaryotic cells in dataset 1 of 7.2 × 10^7^, 7.8 × 10^8^, 1.4 × 10^6^ cells g^−1^ DW soil for qMeTra, NAE_std_, and qRT‐PCR 16S rRNA transcript estimates, respectively.

In comparison, Torsvik et al. (2002) found 4.2 × 10^10^ bacterial cells g^−1^ DW soil by fluorescent microscopy of DAPI stained DNA, which was also reported for prairie, forest and agricultural soils (10^10^ cells g^−1^ DW soil); (Portillo et al. 2013; Torsvik et al. 2002) and the text book states around 10^8^ to 10^9^ colony forming units g^−1^ soil (Metting 1993).

To complicate matters further, microbial carbon and nitrogen extractions from soil come with their own necessary set of corrections for incomplete extraction and biomass conversion factors; thus, we applied standard extraction corrections (see Section 2) and assumed each to represent 50% (Bratbak and Dundas 1984) and 14% (Neidhardt et al. 1990) of a cell's weight, respectively.

Consequently, resulting biomass estimates ranged from 0.2 μg DW biomass g^−1^ DW soil (qRT‐PCR, Table 2) to 2.2 mg DW biomass g^−1^ DW soil (MBC estimates). Range differences between the estimates (Figure 3A) were also carried over in the biomass estimates. Thus, the overall largest and smallest biomass estimates per proxy ranged by a factor of ×11, ×13, ×7, and ×25 for MBC, qRT‐PCR (excl. an outlier), qMeTra and NAE_std_ (excl. an outlier) respectively.

**TABLE 2:** Summary of estimated prokaryotic dry weight biomass (μg g^−1^ DW soil).

Estimates	Summer 2021		Autumn 2021		Winter 2021/22		Spring 2022		Summer 2022	
	B one	B two	B one	B two	B one	B two	B one	B two	B one	B two
NAE_std_	580 ± 111	819 ± 265	259 ± 126	324 ± 272^o^	109 ± 55	66 ± 23	77 ± 38	91 ± 39^o^	81 ± 39	56 ± 5
qMeTra	33 ± 5	38 ± 6	17 ± 5	17 ± 11	17 ± 8	14 ± 3	18 ± 9	22 ± 7	20 ± 9	19 ± 6
16S qRT‐PCR	0.6 ± 0.2	0.8 ± 0.2	0.4 ± 0.2	0.6 ± 0.4	0.3 ± 0.2	0.2 ± 0.1	0.4 ± 0.2	0.6 ± 0.2	0.2 ± 0.1	0.2 ± 0.1
MBC^a^	NA	NA	732 ± 349	873 ± 314	2260 ± 807	1299 ± 548	NA	NA	655 ± 306	1030 ± 701^o^
MBN	NA	NA	411 ± 158	476 ± 209	1260 ± 372	790 ± 276	NA	NA	806 ± 344^o^	412 ± 261^o^

*Note:* MBC and MBN was not available for Summer 2021 or Spring 2022. For Summer 2021, *n* = 4 (biological replicates, i.e., transects), for all other seasons, *n* = 5. Removed outliers are indicated with ‘^o^’ two for NAE_std_ and two for MBN, in these cases *n* = 4. NAE_std_: Estimates based on the ‘nucleic acid extraction standard’. qMeTra: Estimates based on the ‘quantitative metatranscriptomics’ approach Söllinger et al. (2018). 16S qRT‐PCR: Estimates based on qRT‐PCR assays with general 16S EMP primers. MBC and MBN: Estimates based on chloroform fumigation extractions.

^a^ For C‐ and MBN determination a laboratory inconsistency exists, since samples for Autumn 2021 were not sieved prior to chloroform fumigation, while all other samples were initially sieved through 2 mm.

Trying to find validation in the literature is not easy, as very broad generalisations are rarely sufficient. However, Gagelidze et al. (2018) suggested a rule‐of‐thumb that the microbial biomass represents ca. 1% of soil fresh weight (i.e., 10 mg microbial biomass g^−1^ fresh weight soil). Similarly, text book examples state ca. 30 mg wet weight microbial biomass cm^−2^ soil (meaning ca. 7 mg DW biomass, when assuming ca. 0.22 dry: wet weight ratio; Loferer‐Krößbacher et al. 1998; Metting 1993). Although 50% or more of these numbers might represent fungi (Bastida et al. 2021; Verbrigghe et al. 2022), which are not accounted for in our calculation, the examples suggest that all estimates are an underrepresentation of the biomass.

While MBC and MBN estimates were strongly correlated (Pearson's *r* = 0.8, *p* < 0.001), no correlation was found between these and the RNA based estimates. One major discrepancy was the peak of estimated biomass in autumn seen for qRT‐PCR, qMeTra and NAE_std_ estimates, whereas MBC and MBN estimates peaked in winter. From the annotated SSU rRNA profiles, we observed that the relative abundance of *Eukaryota* differed significantly between samples ‘B one’ and ‘B two’ during winter (from 8% to 4%, respectively, Data S6), which likely affects the biomass of these samples and may explain the high values for especially MBC in ‘Winter B one’ samples. Discrepancies were, however, also seen between MBC and MBN estimates, which, for example, differed in the between‐sample pattern for Summer 2022 (Table 2). Possible explanations for these discrepancies include differences in the temporal dynamics of, for example, the presence of carbon storage compounds like polyhydroxybutyrate (PHB) and glucose, as well as variations in cell numbers, influencing MBC, MBN, and NA‐based estimates differently (Bölscher et al. 2024).

**FIGURE 4:**
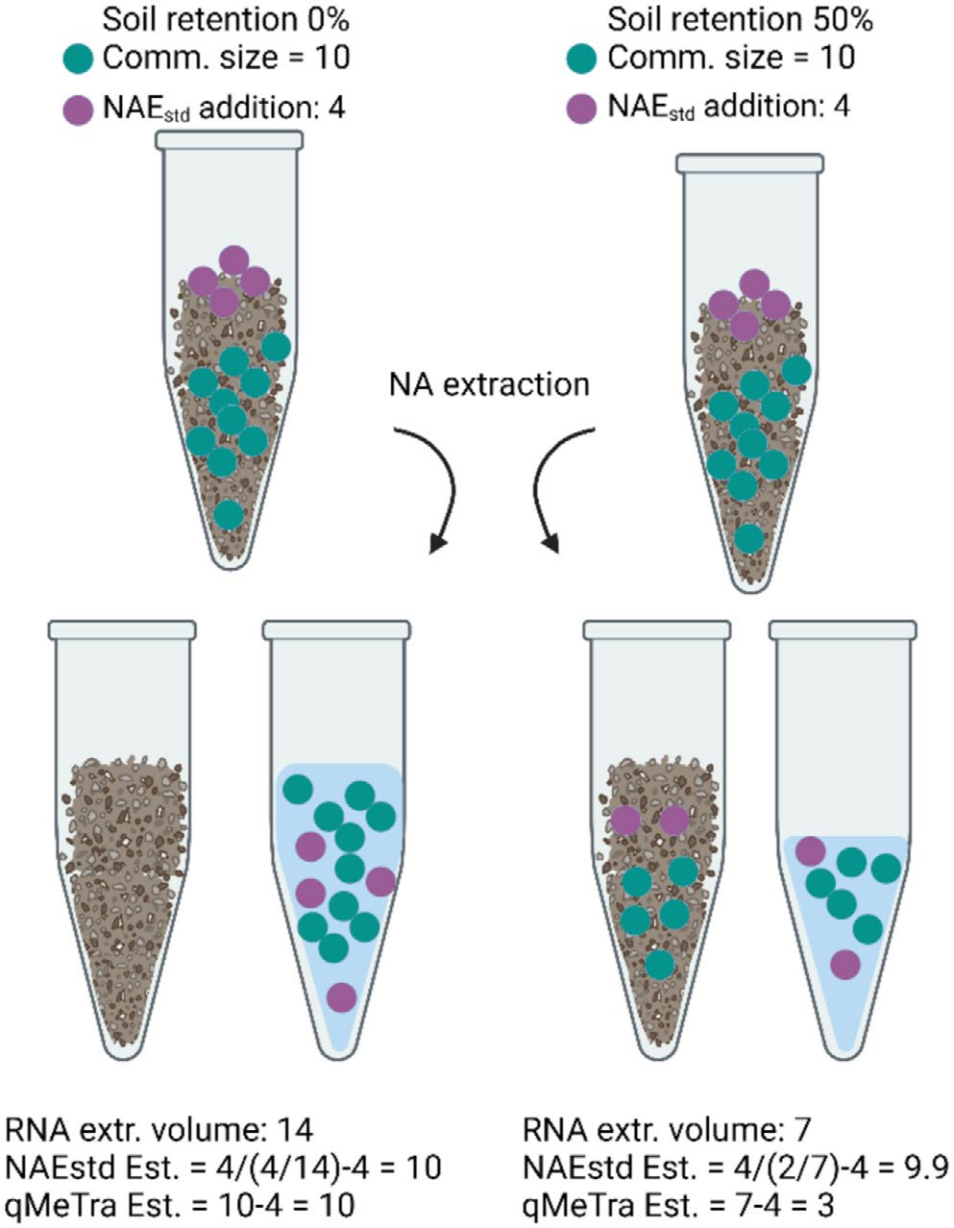
Conceptual drawing of how changes in soil RNA retention strength can result in false community size estimates for qMeTra (and qRT‐PCR), while NAE_std_ estimates will not be affected.

Finally, simultaneous changes in microbial community size and soil NA retention could potentially mask community dynamics and lead to erroneous community estimates for qRT‐PCR and qMeTra, as illustrated in the conceptual Figure 4. The chloroform fumigation extraction (MBC and MBN extraction) is also sensitive to the adsorption strength of the soil and while it cannot be ruled out completely, we would not expect any inhibition of lysis at the given ratio and incubation time. Similarly, cation exchange capacity (CEC), a proxy for the adsorption strength of soils, increases with increasing surface area and water holding capacity. The soil surface area is mostly determined from the amounts of soil organic matter (SOM) and clay content, and although significantly different levels of dissolved organic carbon g^−1^ DW soil (DOC), total carbon and water content were seen across seasons in the analysed samples (Data S6), an effect on the CEC is not expected.

## 4 Concluding Remarks

How much carbon will be released from soil into the atmosphere as a result of global warming? A critical question. To answer it, we need to know how many microorganisms are present and what activities they are performing at any given time in a given soil. Metatranscriptomics provides a way to identify the vast microorganismal and viral diversity present in soil, and gene expression data provide insights into the activity of these organisms. However, true data validation of the quantities—and thereby assessing effect size—is not trivial, since it necessitates correct and certain knowledge about the numbers, sizes and weights of all microorganisms in a given soil sample at a given point in time. This was the core of the scientific challenge of the question and of this article. Including an internal standard (NAE_std_) is a first step towards utilising the full potential of the increasing amount of community information based on molecular data, and the integration of soil (as well as other) microbial community profiles in large‐scale carbon models (Bradford et al. 2016; Filser et al. 2016) Still, it is clear that, in order to make metatranscriptomics fully quantitative, additional efforts, particularly with respect to validation, are needed.

## Author Contributions

M.B.D., A.S., A.T.T. and T.U. designed the study. M.B.D., A.S., A.T.T., L.A., C.G., S.B. and B.D.S. took part in one or more sampling campaigns. S.B., V.G. and M.S. extracted RNA from soil (dataset 1 and 2) and pure cultures and constructed metatranscriptomic libraries. M.S., M.W. and H.W. did (RT)qPCR (dataset 1 and 2). C.S., E.W. and T.P. provided *S. solfataricus* culture pellets, RNA extracts and sequenced transcriptomes (dataset 4). C.G. and A.R. did MBC, MBN, DOC, TC and water content extractions for partial dataset 2. C.J. and A.W.K. were responsible for finalising and sequencing metatranscriptomic libraries (dataset 1 and 2). K.J.H., N.N., A.S., L.A., M.W. and M.B.D. did bioinformatics. M.B.D. evaluated the data and wrote the manuscript, aided by all co‐authors.

## Conflicts of Interest

The authors declare no conflicts of interest.

## Supporting information


**Data S1.** Growth and extraction protocols *Saccharolobus solfataricus*.

**Data S2.** Classification, evaluation and removed of NAE_std_ sequences.

**Data S3.** Relative abundance of *Eukaryota* for ‘AMP’ and ‘nonAMP’ libraries.

**Data S4.** Sequence counts and relative abundance of NAE_std_ reads.

**Data S5.** Bioanalyzer profiles for all RNA extractions.

**Data S6.** Context data relevant for the estimated biomasses.

## Data Availability

Raw sequencing files are available from NCBI, BioProject: PRJNA1130687 (dataset 1 and dataset 2), PRJNA1099624 (dataset4).
