# Supplementary material for: Quantifying Soil Microbiome Abundance by Metatranscriptomics and Complementary Molecular Techniques—Cross‐Validation and Perspectives
Source: Mol Ecol Resour. 2025 Jun 3;25(7):e14130. doi: 10.1111/1755-0998.14130 (PMC12415835; doi:10.1111/1755-0998.14130)
Supplement: Supplementary file 1 — Data S1. Growth and extraction protocols Saccharolobus solfataricus. [file MEN-25-e14130-s006.pdf]

Supplement S1 growth- and extraction protocols *S. solfataricus*

Growing culture condition

S. solfataricus M18, an uracil–auxotrophic derivate of P1 (DSM 1616, ATCC 35091) was grown at 78 °C, pH 3, shaking at 170rpm in Brock medium (Brock et al., 1972) supplemented with 0.2% (+) D-Sucrose (Serva) (w/v) and 0.1% tryptone (Roth) (w/v). Untransformed cells were initially grown with 0.0125mg/ml Uracil (Sigma-Aldrich). For transfection, cells were mixed with the pDEST-MJ-miniCR, a shuttle vector based on the virus SSV1 of *Sulfolobus shibatae* (Jonuscheit et al., 2003) carrying a miniCRISPR array with non-targeting spacers (Zebec et al., 2014). Electroporation, inverse plaque assay and cultivation of transfectants was carried out as described previously (Zink et al., 2019). Growth of *S. solfataricus* cells was monitored at OD of 600 nm (Beckam Coulter, DU 800 Spectrophotometer), once transfected cultures reached an OD_{600} of 0.2, 5-50ml of cells were harvested via centrifugation at 2000 × g and 4 °C for 15 min and cell pellets were stored at -70°C.

RNA extracts from *S. solfataricus*

Frozen pellets were thawed on ice for 10 minutes before RNA was extracted with the mirVanaTM kit (Ambion) according to the manufacturer's instructions, pellets were resuspended in 600µl of the Lysis/Binding buffer. RNA was eluted in 100µl DEPC-water 0.1% (Roth), and concentration and potential phenol contamination was measured via NanoDrop (ND-1000, PeqLab). RNA was DNase treated (TURBO DNA-free kit, ThermoFisher Scientific) according to the manufacturers instructions, and finally RNA concentration was measured via Qubit™ RNA High Sensitivity (HS) Assay Kit (ThermoFisher Scientific) and RNA was stored at -70°C.

Qualitative analysis of the RNA extracts was performed using Agilent RNA 6000 Nano Kit with the 2100 Bioanalyzer system by Agilent Technologies.

Literature for Supplement S1

Brock, T. D., Brock, K. M., Belly, R. T., & Weiss, R. L. (1972). Sulfolobus: A new genus of sulfur-oxidizing bacteria living at low pH and high temperature. *Archiv for Mikrobiologie*, *84*(1), 54–68. https://doi.org/10.1007/BF00408082

Jonuscheit, M., Martusewitsch, E., Stedman, K. M., & Schleper, C. (2003). A reporter gene system for the hyperthermophilic archaeon Sulfolobus solfataricus based on a selectable and integrative shuttle vector. *Molecular Microbiology*, *48*(5), 1241–1252. https://doi.org/10.1046/j.1365-2958.2003.03509.x

Zebec, Z., Manica, A., Zhang, J., White, M. F., & Schleper, C. (2014). CRISPR-mediated targeted mRNA degradation in the archaeon Sulfolobus solfataricus. *Nucleic Acids Research*, *42*(8), 5280–5288. https://doi.org/10.1093/nar/gku161

Zink, I. A., Pfeifer, K., Wimmer, E., Sleytr, U. B., Schuster, B., & Schleper, C. (2019). CRISPR-mediated gene silencing reveals involvement of the archaeal S-layer in cell division and virus infection. *Nature Communications*, *10*(1). https://doi.org/10.1038/s41467-019-12745-x
