# Supplementary material for: Quantifying Soil Microbiome Abundance by Metatranscriptomics and Complementary Molecular Techniques—Cross‐Validation and Perspectives
Source: Mol Ecol Resour. 2025 Jun 3;25(7):e14130. doi: 10.1111/1755-0998.14130 (PMC12415835; doi:10.1111/1755-0998.14130)
Supplement: Supplementary file 2 — Data S2. Classification, evaluation and removed of NAEstd sequences. [file MEN-25-e14130-s003.pdf]

Supplement S2 – SSU and non-rRNA classification

SSU rRNA classification from culture transcriptomes of *S. solfataricus,* dataset 4.

Table S1 Overview of recovery and classification of SSU rRNA sequences from 4x pure culture transcriptomes of *S. solfataricus* (dataset 4) used as nucleic acid extraction standard (NAE_{std}) in the environmental soil samples.

	**Sol.1**	**Sol.2**	**Sol.3**	**Sol.4**
**SSU rRNA reads**				
Full sample size	13,247,446	13,117,904	11,824,782	13,548,186
Total SSU rRNA	2,040,802	2,119,861	2,119,861	2,023,982
**Taxonomic classification**				
Archaea	2,039,030 (99.9%)	2,117,887 (99.9%)	2,735,170 (99.8%)	2,022,625 (99.9%)
Crenarchaeota				
Thermoprotei (class)	2,038,904 (99.9%)	2,117,691 (99.9%)	2,735,127 (99.8%)	2,022,504 (99.9%)
Saccharolobus sp.	1,674,948 (82.1%)	1,796,723 (84.8%)	2,283,360 (83.3%)	1,667,399 (82.4%)
Bacteria	1,771 (< 0.01%)	1,991 (< 0.01%)	1,991 (< 0.01%)	1,360 (< 0.01%)
Proteobacteria	1,757	1,969	1,969	1,355
E. coli	1,732	1,936	1,936	1,308
Eukaryota	1	5	5	2

Non-rRNA classification from culture transcriptomes of *S. solfataricus,* dataset 4.

Table S2 Overview of recovery and classification of mRNA reads from the pure culture transcriptomes of *S. solfataricus.*

**Putative mRNA reads and viruses**				
	**Sol.1**	**Sol.2**	**Sol.3**	**Sol.4**
Total non-rRNA	4,559,126	4,353,802	3,615,192	4,039,964
Identified mRNA/vira	4,449,244	4,254,923	3,532,029	3,944,095
**Taxonomic classification**				
Crenarchaeota	4,442,576 (99.9%)	4,248,107 (99.8%)	3,526,427 (99.8%)	3,937,871 (99.8%)
Proteobacteria	239 (0.05‰)	151 (0.04‰)	226 (0.06‰)	161 (0.04‰)
Taleaviricota (virus)	3,252 (0.07%)	3,048 (0.07%)	2,387 (0.07%)	3,232 (0.08%)
Other	113 (0.03‰)	82 (0.02‰)	107 (0.03‰)	80 (0.02‰)

SSU rRNA classification from natural reference metatranscriptomic datasets 'forhot', dataset 3.

Table S3 Overview of recovery and classification of SSU rRNA sequences from the 4x reference metatranscriptomic datasets 'forhot' 2016.

	**MTW-4-ET (F1)**	**LTW-4-ET (F2)**	**LTW-4-AT (F3)**	**MTW-4-AT (F4)**
**Full sample size**	116,028,701	117,877,779	114,038,445	110,962,314
**Total SSU rRNA**	15,100,969	20,269,744	18,160,122	13,980,112
**% SSU of full sample**	13.0%	17.2%	15.9%	12.6%
**Matching classification of SSU rRNA**				
**Archaea**	33,086 (0.2%)	4,546 (< 0.1%)	7,816 (< 0.1%)	21,712 (0.2%)
Crenarchaeota	31,538 (95.3% of Arc)	4,253 (93.6% of Arc)	6,494 (83.1% of Arc)	21,460 (98.8% of Arc)
Nitrososphaeria (class)	31,495 (99.9% of Cre)	4, 216 (99.1% of Cre)	6,492 (> 99.9% of Cre)	21,457 (> 99.9% of Cre)
Thermoprotei (class)	0	0	0	0
**Bacteria**	12,173,017 (80.6%)	16,402,063 (80.9%)	13,631,393 (75.1%)	11,605,177 (83.0%)
Proteobacteria	1,627,320 (13.4% of Bac)	2,454,188 (15.0% of Bac)	2319169 (17.0% of Bac)	1,850,382 (15.9% of Bac)
Enterobacteriaceae	1,991 (0.16‰ of Bac)	1,463 (0.16‰ of Bac)	106 (0.01‰ of Bac)	2,571 (0.22‰ of Bac)
E. coli	61 (< 0.01‰ of Bac)	45 (< 0.01‰ of Bac)	61 (< 0.01‰ of Bac)	61 (< 0.01‰ of Bac)
**Eukaryota**	2,894,866 (19.2%)	3,863,135 (19.1%)	4,520,913 (24.9%)	2,353,223 (16.8%)

SSU rRNA classification of *in silico* mock community

Table S4 Overview of recovery and classification of non-rRNA sequences from the mixed mock samples of dataset 3 and 4.

**Mock samples for phyloFLASH pipeline**	**Reads**
**Full sample size** (4x 200k 'NAE$_{std}$' + 4x 200k 'forhot')	1,600,000
**Identified SSU rRNA** originating from *S. solfataricus*	177,310
**Identified SSU rRNA** originating from 'forhot' samples	208,183
**Total identified SSU rRNA reads**	385,493
**Total classified (LCA) SSU rRNA reads**	257,173
**% SSU rRNA** reads of total sample	24.1%
**Classification of SSU rRNA reads of dataset 4 from mock sample (*S. solfataricus*)**	
**Taxonomy**	**Read (% or ‰ reads)**
**Archaea**<br>*Crenarchaeota*<br>*Thermoprotei* (class)<br>*Bathyarchaeia* (class)	 177.119 (99.9% of NAE$_{std}$ reads)<br>177.118 (99.8% of NAE$_{std}$ reads)<br>1
**Bacteria**<br>*Proteobacteria*<br>*Escherichia-Shigella*<br>*E. coli*<br>Other genera of *Enterobacteriaceae* (family)	 191 (0.1% of NAE$_{std}$ reads)<br>176 (92% of *Proteobacteria*)<br>170 (89% of *Proteobacteria*)<br>14 (7.3% of *Proteobacteria*, 0.08‰ of NAE$_{std}$ reads)
**Eukaryota**	0
**Matching classification of SSU rRNA reads of dataset 3 from mock sample ('forhot' 2016)**	
**Taxonomy**	**Reads (% or ‰ reads)**
**Archaea**<br>*Crenarchaeota*<br>*Thermoprotei* (class)	 157 (0.8‰ of 'forhot' reads)<br>0
**Bacteria**<br>*Proteobacteria*<br>*Escherichia-Shigella*<br>Other genera of *Enterobacteriaceae* (family)	 22,003 (10.6% of 'forhot' reads)<br>9 (0.04% of *Proteobacteria*)<br>23 (0.1% of *Proteobacteria,* 0.01% of 'forhot' reads)

Reference databases for non-rRNA mock samples

We build reference databases of both the four transcriptomes presented in this study as well as from publicly available genomes of *S. solfataricus* listed her:

Table S5

Strain	BioSample	BioProject	Assembly	Size (Mb)	GC%	Scaffolds	Release Date	used in the study?
P1	SAMEA3920617	PRJEB13308	GCA_900079115.1	3.03402	35.8	2942	2016-04-11T00:00:00Z	yes
POZ149	SAMN14482681	PRJNA616127	GCA_015654385.1	3.02482	35.8	2877	2020-11-23T00:00:00Z	yes
SULA	SAMN03452280	PRJNA279935	GCA_000968435.2	2.72731	35.9	2678	2015-04-02T00:00:00Z	yes
SUL120	SAMN06006254	PRJNA352873	GCA_003852115.1	2.70751	35.8	2638	2018-11-29T00:00:00Z	yes
SULG	SAMN05896255	PRJNA347847	GCA_003852135.1	2.66897	35.8	2638	2018-11-29T00:00:00Z	yes
SARC-H	SAMN05896744	PRJNA347851	GCA_003852195.1	2.66897	35.8	2638	2018-11-29T00:00:00Z	yes
SARC-I	SAMN05896745	PRJNA347852	GCA_003852215.1	2.66897	35.8	2638	2018-11-29T00:00:00Z	yes
SARC-O	SAMN05896748	PRJNA347857	GCA_003852095.1	2.65914	35.8	2625	2018-11-29T00:00:00Z	yes
SARC-N	SAMN05896747	PRJNA347855	GCA_003852175.1	2.65814	35.8	2624	2018-11-29T00:00:00Z	yes
P2	SAMN02603227	PRJNA108	GCA_000007005.1	2.99225	35.8	2865	2001-10-03T00:00:00Z	yes
98/2	SAMN00001854	PRJNA33849	GCA_000175555.1	2.68047	35.2	506	2009-07-20T00:00:00Z	no [1]
SULM	SAMN05896746	PRJNA347854	GCA_003852155.1	2.65814	35.8	2625	2018-11-29T00:00:00Z	yes
SARC-B	SAMN03452281	PRJNA279939	GCA_000968355.2	2.72731	35.9	2678	2015-04-02T00:00:00Z	yes
SARC-C	SAMN03395044	PRJNA277733	GCA_000968395.2	2.72731	35.9	2678	2015-04-02T00:00:00Z	yes

[1] Record suppressed due to contamination.

Non-rRNA classification of *in silico* mock community

Table S6 Overview of recovery of spiked sequences of the non-rRNA sequences from the mixed mock samples of dataset 3 and 4. Test mock sample size was 5 mio. sequences per sample and tested spiking -levels were 0.1%, 1% and 10% of “dataset 4” (*S. solfataricus*) sequences.

File ID	spiked	% spiked nominal	total # of sequences	# of spike sequences	# of forhot sequences	% spiked real	# SPIKE seq in output	% SPIKE seq recovered	# FORHOT seq in output	% FORHOT seq in output
LTW_1_AT_non_rRNA_10_pc_spiked_randomO.fasta	yes	10	4998301	499451	4498850	9.99	499396	99.99	11506	0.26
LTW_1_ET_non_rRNA_10_pc_spiked_randomO.fasta	yes	10	4998498	499648	4498850	10.0	499590	99.99	15540	0.35
LTW_2_AT_non_rRNA_10_pc_spiked_randomO.fasta	yes	10	4998224	499374	4498850	9.99	499304	99.99	8879	0.20
LTW_2_ET_non_rRNA_10_pc_spiked_randomO.fasta	yes	10	4998389	499539	4498850	9.99	499470	99.99	13045	0.29
LTW_3_AT_non_rRNA_10_pc_spiked_randomO.fasta	yes	10	4998050	499202	4498848	9.99	499156	99.99	11059	0.25
LTW_3_ET_non_rRNA_10_pc_spiked_randomO.fasta	yes	10	4998850	500000	4498850	10.0	499950	99.99	11958	0.27
LTW_4_AT_non_rRNA_10_pc_spiked_randomO.fasta	yes	10	4998674	499824	4498850	10.0	499770	99.99	12193	0.27
LTW_4_ET_non_rRNA_10_pc_spiked_randomO.fasta	yes	10	4997786	498936	4498850	9.98	498869	99.99	14082	0.31
LTW_1_AT_non_rRNA_1_pc_spiked_randomO.fasta	yes	1	4999919	49987	4949932	1.00	49981	99.99	12662	0.26
LTW_1_ET_non_rRNA_1_pc_spiked_randomO.fasta	yes	1	4999932	50000	4949932	1.00	49996	99.99	17112	0.35
LTW_2_AT_non_rRNA_1_pc_spiked_randomO.fasta	yes	1	4999856	49924	4949932	1.00	49917	99.99	9762	0.20
LTW_2_ET_non_rRNA_1_pc_spiked_randomO.fasta	yes	1	4999816	49884	4949932	1.00	49880	99.99	14360	0.29
LTW_3_AT_non_rRNA_1_pc_spiked_randomO.fasta	yes	1	4999757	49826	4949931	1.00	49820	99.99	12148	0.25
LTW_3_ET_non_rRNA_1_pc_spiked_randomO.fasta	yes	1	4999535	49603	4949932	0.99	49596	99.99	13131	0.27
LTW_4_AT_non_rRNA_1_pc_spiked_randomO.fasta	yes	1	4999932	50000	4949932	1.00	49994	99.99	13453	0.27
LTW_4_ET_non_rRNA_1_pc_spiked_randomO.fasta	yes	1	4999752	49820	4949932	1.00	49814	99.99	15572	0.31
LTW_1_AT_non_rRNA_0.1_pc_spiked_randomO.fasta	yes	0.1	5000000	5000	4995000	0.10	5000	100.00	12767	0.26

LTW_1_ET_non_rRNA_0.1_pc_spiked_randomO.fasta	yes	0.1	5000000	5000	4995000	0.10	4992	99.84	9848	0.20
LTW_2_AT_non_rRNA_0.1_pc_spiked_randomO.fasta	yes	0.1	4999989	4989	4995000	0.10	4988	99.98	12262	0.25
LTW_2_ET_non_rRNA_0.1_pc_spiked_randomO.fasta	yes	0.1	4999993	4993	4995000	0.10	4992	99.98	13568	0.27
LTW_3_AT_non_rRNA_0.1_pc_spiked_randomO.fasta	yes	0.1	4999985	4985	4995000	0.10	4984	99.98	17261	0.35
LTW_3_ET_non_rRNA_0.1_pc_spiked_randomO.fasta	yes	0.1	4999953	4953	4995000	0.10	4950	99.94	14515	0.29
LTW_4_AT_non_rRNA_0.1_pc_spiked_randomO.fasta	yes	0.1	5000000	5000	4995000	0.10	5000	100.00	13243	0.27
LTW_4_ET_non_rRNA_0.1_pc_spiked_randomO.fasta	yes	0.1	5000000	5000	4995000	0.10	5000	100.00	15695	0.31
LTW_1_AT_non_rRNA_spike_free_5mill.fasta	no	0	5000000	0	5000000	0	0	0	12776	0.26
LTW_1_ET_non_rRNA_spike_free_5mill.fasta	no	0	5000000	0	5000000	0	0	0	17279	0.35
LTW_2_AT_non_rRNA_spike_free_5mill.fasta	no	0	5000000	0	5000000	0	0	0	9856	0.20
LTW_2_ET_non_rRNA_spike_free_5mill.fasta	no	0	5000000	0	5000000	0	0	0	14532	0.29
LTW_3_AT_non_rRNA_spike_free_5mill.fasta	no	0	4999998	0	4999998	0	0	0	12274	0.25
LTW_3_ET_non_rRNA_spike_free_5mill.fasta	no	0	5000000	0	5000000	0	0	0	13254	0.27
LTW_4_AT_non_rRNA_spike_free_5mill.fasta	no	0	5000000	0	5000000	0	0	0	13577	0.27
LTW_4_ET_non_rRNA_spike_free_5mill.fasta	no	0	5000000	0	5000000	0	0	0	15708	0.31
**AVERAGE**								**99.98%**		**0.27%**
