# Supplementary material for: Quantifying Soil Microbiome Abundance by Metatranscriptomics and Complementary Molecular Techniques—Cross‐Validation and Perspectives
Source: Mol Ecol Resour. 2025 Jun 3;25(7):e14130. doi: 10.1111/1755-0998.14130 (PMC12415835; doi:10.1111/1755-0998.14130)

Supplement S3

Abundance differences among SSU rRNA assignments to Eukaryota of AMP and nonAMP libraries.

Phyla rank.

Almost all major eukaryotic groups have an increased abundance in AMP libraries (green).

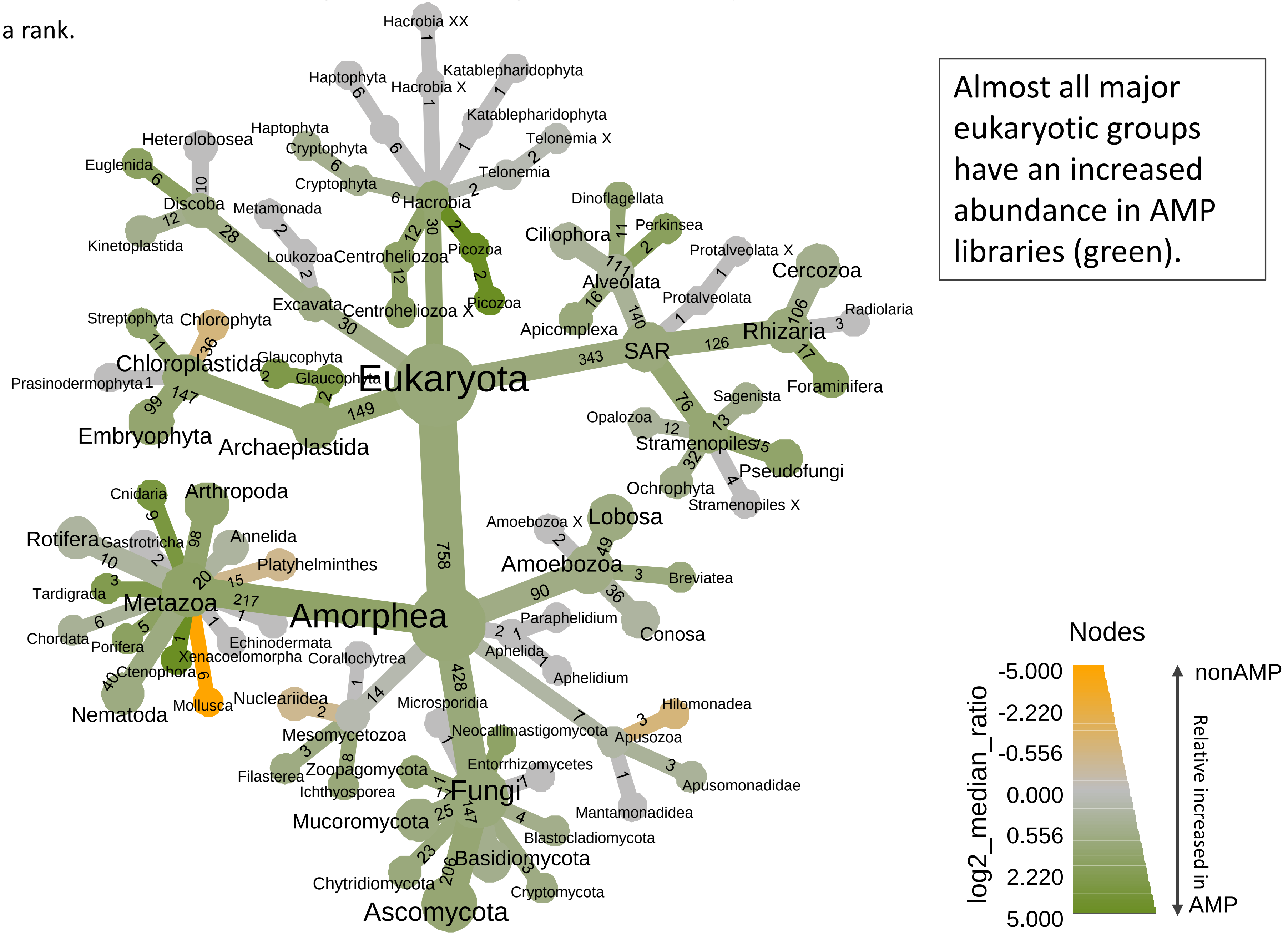

Filter: n > 3. Color represent significant differences (fdr-corrected, $p < 0.05$)

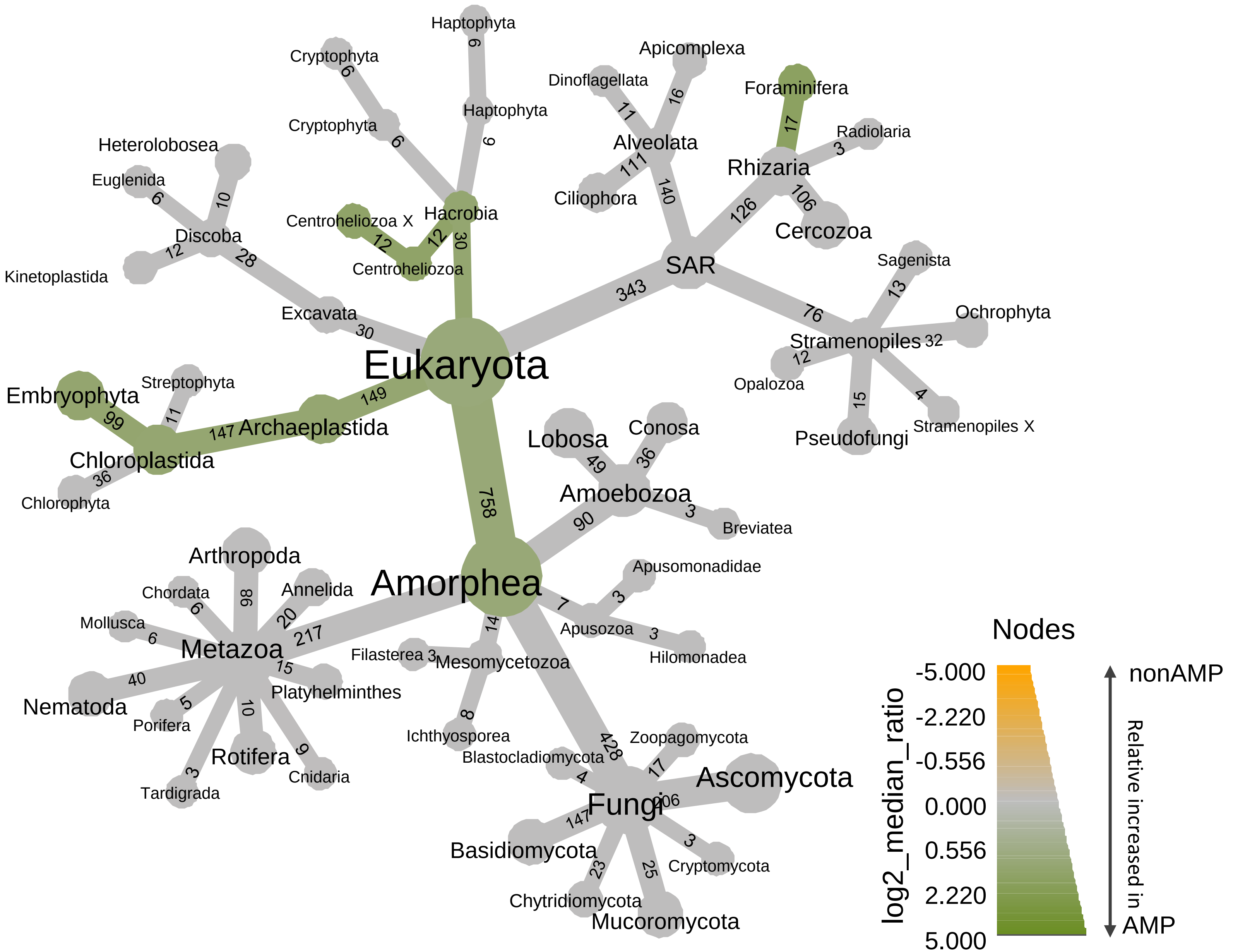

Supplement: Supplementary file 3 — Data S3. Relative abundance of Eukaryota for ‘AMP’ and ‘nonAMP’ libraries. [file MEN-25-e14130-s005.pdf]
