# Supplementary material for: Quantifying Soil Microbiome Abundance by Metatranscriptomics and Complementary Molecular Techniques—Cross‐Validation and Perspectives
Source: Mol Ecol Resour. 2025 Jun 3;25(7):e14130. doi: 10.1111/1755-0998.14130 (PMC12415835; doi:10.1111/1755-0998.14130)
Supplement: Supplementary file 4 — Data S4. Sequence counts and relative abundance of NAEstd reads. [file MEN-25-e14130-s004.pdf]

Supplement S4 – Sequence counts and relative abundance of NAE_{std} reads.

		Sample info			sequenced data				
	seq ID	sample	[ng] total extracted RNA after purification	g input soil wet weight	total size of sample (R1)	theory%	Expected SSU-fraction (32%)	Actaual SSU rRNA reads SortMeRNA (phyloFlash)	diff AMP:nonAMP
AMP	S13	GN-1-A 1	832	0.56	18,373,504	32	5,879,521	3,606,029	41%
	S14	GN-1-A 2	612	0.53	17,344,300	32	5,550,176	3,348,276	31%
	S15	GN-1-A 3	492	0.52	12,816,477	32	4,101,273	2,481,885	23%
	S1	GN-1-E 1	387	0.56	23,032,679	32	7,370,457	4,875,592	52%
	S2	GN-1-E 2	414	0.54	20,846,342	32	6,670,829	3,055,452	30%
	S3	GN-1-E 3	408	0.53	18,502,466	32	5,920,789	2,707,073	22%
	S4	GO-4-A 1	780	0.51	21,935,449	32	7,019,344	3,477,673	31%
	S5	GO-4-A 2	230	0.55	22,394,350	32	7,166,192	3,570,772	32%
	S6	GO-4-A 3	770	0.55	20,643,356	32	6,605,874	3,279,646	28%
	S16	GO-4-E 1	760	0.51	20,695,860	32	6,622,675	3,805,729	37%
	S17	GO-4-E 2	1280	0.5	15,126,255	32	4,840,402	2,666,297	27%
	S18	GO-4-E 3	560	0.5	19,806,048	32	6,337,935	3,560,266	38%
nonAMP	S19	GN-1-A 1	832	0.56	16,048,126	32	5,135,400	8,875,285	
	S20	GN-1-A 2	612	0.53	19,846,931	32	6,351,018	10,668,310	
	S21	GN-1-A 3	492	0.52	19,590,777	32	6,269,049	10,586,352	
	S7	GN-1-E 1	387	0.56	16,808,922	32	5,378,855	9,391,691	
	S8	GN-1-E 2	414	0.54	18,767,380	32	6,005,562	10,318,139	
	S9	GN-1-E 3	408	0.53	19,729,327	32	6,313,385	12,035,626	
	S10	GO-4-A 1	780	0.51	19,192,588	32	6,141,628	11,043,938	
	S11	GO-4-A 2	230	0.55	20,169,640	32	6,454,285	11,199,597	
	S12	GO-4-A 3	770	0.55	21,019,110	32	6,726,115	11,782,410	
	S22	GO-4-E 1	760	0.51	18,480,306	32	5,913,698	10,402,417	
	S23	GO-4-E 2	1280	0.5	17,691,750	32	5,661,360	9,938,920	
	S24	GO-4-E 3	560	0.5	16,748,695	32	5,359,582	9,408,831	
				avarage	18,983,777		AMP mean	3,369,558	
				sd	2,384,981		AMPS sd	634,713	
							nonAMP mean	10,470,960	
				sum	455,610,638		nonAMP sd	964,355	

NextSeqID	total RNA [ng]	ngRNADWsoil	totRNA	SSU	LSUmRNA	nonrRNA	%nonrRNA	%Thermoprotei sp.
S388	1150	3647	12353515	2366842	2862241	338753	2.7	0.12
S392	1580	3836	10912974	2651225	3502066	248820	2.3	0.18
S389	1120	4509	13913985	2816465	3314055	455983	3.3	0.14
S393	1140	5366	14228645	4198363	4752976	409055	2.9	0.09
S390	1290	5220	10796591	2033473	2426954	264294	2.4	0.07
S394	1480	3621	11547445	2278862	2860371	272197	2.4	0.11
S391	1880	4702	11548773	2727198	3522549	320260	2.8	0.10
S395	1270	4196	13274314	3375313	4488452	373827	2.8	0.15
S396	970	3102	14162546	2456368	3215845	257526	1.8	0.21
S401	1460	2554	19145978	3195776	4042596	238218	1.2	0.28
S397	710	1872	15610694	2621547	3314636	267588	1.7	0.26
S402	570	2327	11916899	2090220	2759160	236826	2.0	0.23
S398	575	1085	14960099	2672571	3357744	297914	2.0	2.16
S403	420	5463	12392333	2355698	3049058	263116	2.1	0.10
S399	785	1879	13065937	2522643	2990600	252214	1.9	0.38
S404	346	1387	11180445	2409391	3020878	287505	2.6	0.25
S400	369	1046	12346904	1986944	3579766	1347524	10.9	0.35
S405	424	1495	12162230	3216473	3729976	563722	4.6	1.63
S426	1500	3955	15521417	2967847	3703883	260710	1.7	0.66
S409	520	2562	14117424	3946720	4811111	348045	2.5	0.49
S406	800	2186	12842864	3069240	3707928	284644	2.2	1.50
S410	540	1608	14242171	3605226	4561390	512136	3.6	0.86
S407	505	1807	17410473	3638207	4377274	336401	1.9	1.23
S411	372	1602	11470465	3075884	3968948	341501	3.0	1.60
S427	930	6704	12282853	1650258	2025010	86826	0.7	0.32
S428	615	3405	12448052	2534112	3153376	240458	1.9	0.44
S408	555	1873	10359245	2065649	3395151	335511	3.2	1.09
S429	875	5645	16663831	3789614	4930850	1001503	6.0	0.99
S430	890	3008	14474575	2687229	3370985	390406	2.7	0.89
S439	880	3794	15702831	3046829	3496450	335706	2.1	0.68
S431	1100	1646	11847922	2417664	2969517	325939	2.8	1.90
S434	*650*	879	*12074235*	*2146628*	*2542295*	*247941*	*2.1*	*49.84*
S432	487	1981	17467295	3876503	4534321	597452	3.4	0.94
S435	1160	3666	14572747	2581024	3208307	310267	2.1	0.72
S438	635	3975	16377674	3340238	3857623	463975	2.8	0.77
S440	516	3090	15723540	3265673	3769936	459343	2.9	0.48
S433	260	2104	16571832	2238377	6509210	3020763	18.2	1.72
S441	1040	3065	16733734	3375178	3922743	383705	2.3	0.84
S436	1150	1761	14514748	2915147	3259975	375743	2.6	2.41
S445	525	3413	15177251	3035475	3277952	352090	2.3	0.57
S437	815	2674	15883280	2585826	3159174	353257	2.2	2.00
S446	880	2412	14610599	2624967	3084720	329141	2.3	1.24
S442	780	722	16750623	3498141	4175817	603993	3.6	1.62
S447	765	1918	13800761	2911033	3346851	712562	5.2	1.35
S443	680	2859	13037722	2794818	3072140	358683	2.8	1.45
S448	640	2519	15734370	3428868	3810678	513198	3.3	1.50
S444	249	1798	16512104	3536534	3648077	266179	1.6	1.97
S449	*432*	1521	*13484882*	*2705959*	*3207580*	*718239*	*5.3*	*4.67*
Ave.	831	2.9					3	0.8
sd.	383	1.4					1	0.7

Theoretical abundance of NAEstd in complete RNA extraction:

3.6%

Light grey marks outliers.
