# Supplementary material for: Quantifying Soil Microbiome Abundance by Metatranscriptomics and Complementary Molecular Techniques—Cross‐Validation and Perspectives
Source: Mol Ecol Resour. 2025 Jun 3;25(7):e14130. doi: 10.1111/1755-0998.14130 (PMC12415835; doi:10.1111/1755-0998.14130)
Supplement: Supplementary file 5 — Data S5. Bioanalyzer profiles for all RNA extractions. [file MEN-25-e14130-s002.pdf]

Supplement S5

2100 Bioanalyzer profiles

Dataset 1 – GN-A and -E is synonymous with sample ‘A one’ and ‘A two’ respectively. Likewise, GO-A and -E is synonymous with sample ‘B one’ and ‘B two’.

Electropherogram Summary

Sample 1: GN-1-A 1

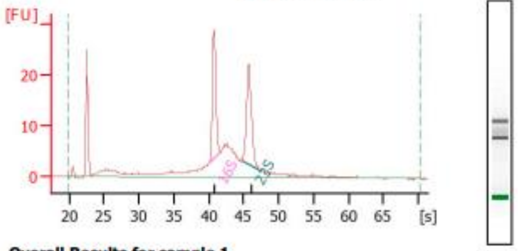

Overall Results for sample 1

RNA Area:	208.0
RNA Concentration:	67 ng/µl
rRNA Ratio [23s / 16s]:	1.0
RNA Integrity Number (RIN):	8.4 (B.02.09)

Fragment table for sample 1

Name	Start Time [s]	End Time [s]	Area	% of total Area
16S	40.06	41.59	35.7	17.2
23S	44.80	47.44	36.9	17.8

Sample 2: GN-1-A 2

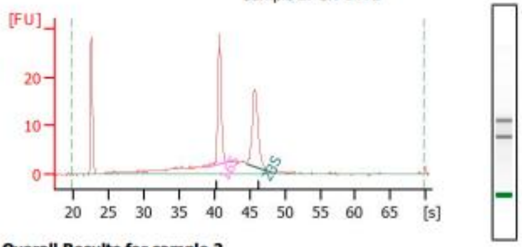

Overall Results for sample 2

RNA Area:	163.1
RNA Concentration:	52 ng/µl
rRNA Ratio [23s / 16s]:	0.9
RNA Integrity Number (RIN):	8.7 (B.02.09)

Fragment table for sample 2

Name	Start Time [s]	End Time [s]	Area	% of total Area
16S	38.83	41.81	37.5	23.0
23S	44.54	48.19	34.5	21.1

Sample 3: GN-1-A 3

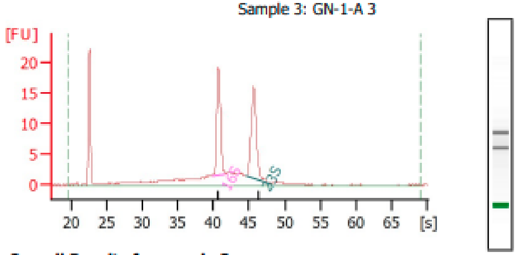

Overall Results for sample 3

RNA Area:	118.7
RNA Concentration:	38 ng/µl
rRNA Ratio [23s / 16s]:	1.1
RNA Integrity Number (RIN):	8.6 (B.02.09)

Fragment table for sample 3

Name	Start Time [s]	End Time [s]	Area	% of total Area
16S	39.84	41.64	25.9	21.8
23S	44.65	48.11	29.0	24.4

Sample 4: GN-1-E 1

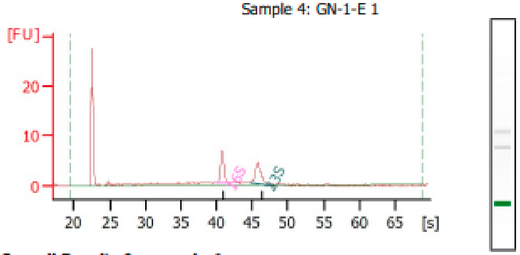

Overall Results for sample 4

RNA Area:	39.4
RNA Concentration:	13 ng/µl
rRNA Ratio [23s / 16s]:	1.0
RNA Integrity Number (RIN):	8.6 (B.02.09)

Fragment table for sample 4

Name	Start Time [s]	End Time [s]	Area	% of total Area
16S	40.01	41.98	8.7	22.1
23S	44.75	48.09	8.6	21.7

Sample 5: GN-1-E 2

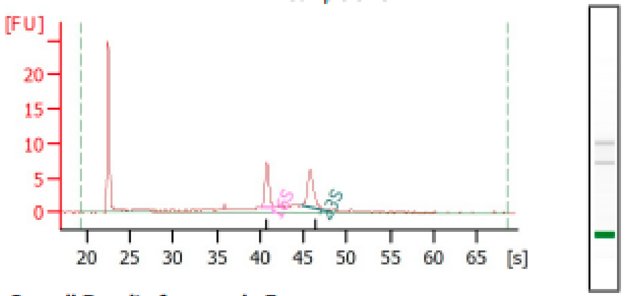

Overall Results for sample 5

RNA Area:	54.3
RNA Concentration:	17 ng/µl
rRNA Ratio [23s / 16s]:	1.1
RNA Integrity Number (RIN):	8.6 (B.02.09)

Fragment table for sample 5

Name	Start Time [s]	End Time [s]	Area	% of total Area
16S	39.98	41.77	8.8	16.2
23S	44.80	48.23	10.1	18.6

Sample 6: GN-1-E 3

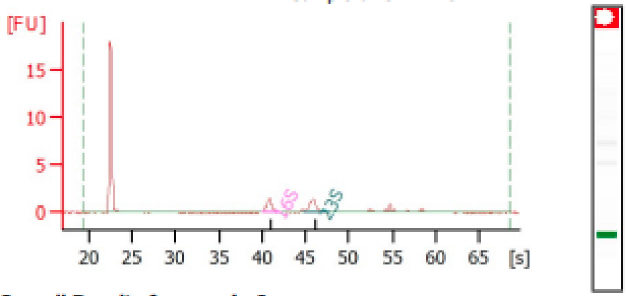

Overall Results for sample 6

RNA Area:	6.7
RNA Concentration:	2 ng/µl
rRNA Ratio [23s / 16s]:	1.1
RNA Integrity Number (RIN):	N/A (B.02.09)

Fragment table for sample 6

Name	Start Time [s]	End Time [s]	Area	% of total Area
16S	39.98	42.02	2.4	35.7
23S	44.85	47.83	2.6	38.7

Sample 7: GO-4-A 1

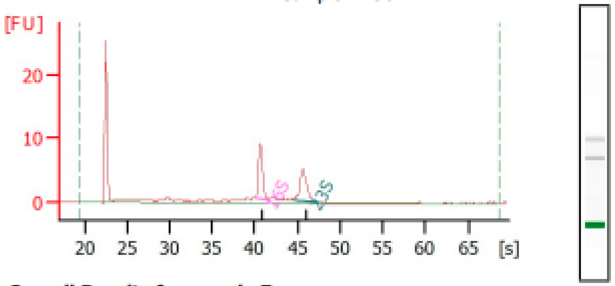

Overall Results for sample 7

RNA Area:	44.1
RNA Concentration:	14 ng/µl
rRNA Ratio [23s / 16s]:	0.9
RNA Integrity Number (RIN):	8.6 (B.02.09)

Fragment table for sample 7

Name	Start Time [s]	End Time [s]	Area	% of total Area
16S	40.02	41.66	11.4	26.0
23S	44.55	47.39	10.0	22.8

Sample 8: GO-4-A 2

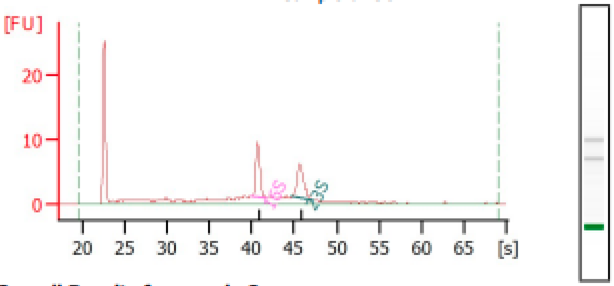

Overall Results for sample 8

RNA Area:	74.8
RNA Concentration:	24 ng/µl
rRNA Ratio [23s / 16s]:	1.0
RNA Integrity Number (RIN):	8.1 (B.02.09)

Fragment table for sample 8

Name	Start Time [s]	End Time [s]	Area	% of total Area
16S	40.04	41.74	11.5	15.4
23S	44.75	47.31	11.0	14.8

Sample 9: GO-4-A 3

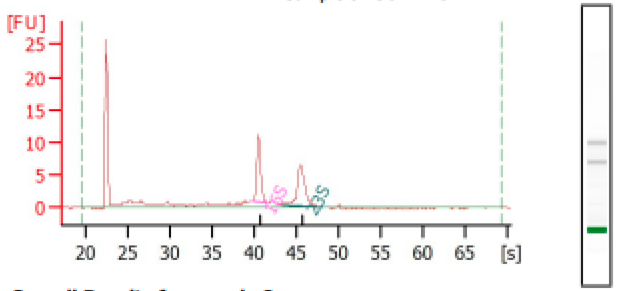

Overall Results for sample 9

RNA Area:	68.2
RNA Concentration:	22 ng/µl
rRNA Ratio [23s / 16s]:	1.1
RNA Integrity Number (RIN):	8.5 (B.02.09)

Fragment table for sample 9

Name	Start Time [s]	End Time [s]	Area	% of total Area
16S	39.97	41.78	13.7	20.0
23S	43.44	48.12	15.6	22.9

Sample 10: GO-4-E

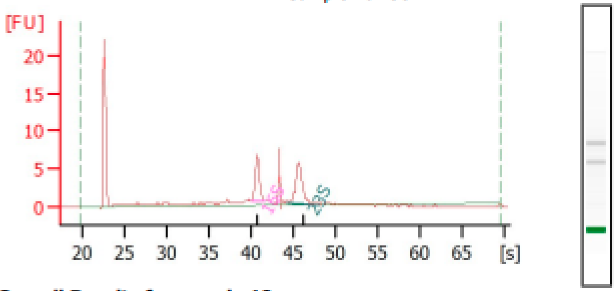

Overall Results for sample 10

RNA Area:	42.6
RNA Concentration:	14 ng/µl
rRNA Ratio [23s / 16s]:	1.2
RNA Integrity Number (RIN):	8 (B.02.09)

Fragment table for sample 10

Name	Start Time [s]	End Time [s]	Area	% of total Area
16S	40.01	41.62	8.5	19.9
23S	44.34	48.03	10.5	24.6

Go-4-E 1

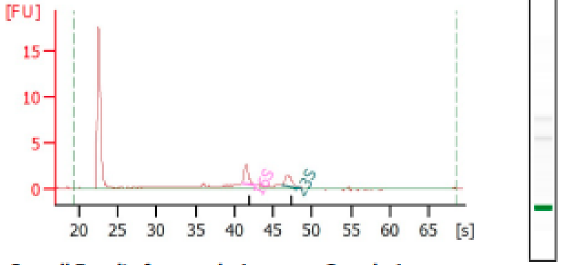

Overall Results for sample 1 : Sample 1

RNA Area:	19.8
RNA Concentration:	19 ng/µl
rRNA Ratio [23s / 16s]:	0.6
RNA Integrity Number (RIN):	7.8 (B.02.09)

Fragment table for sample 1 : Sample 1

Name	Start Time [s]	End Time [s]	Area	% of total Area
16S	40.93	42.86	3.6	18.3
23S	46.24	48.73	2.3	11.6

Go-4-E 2

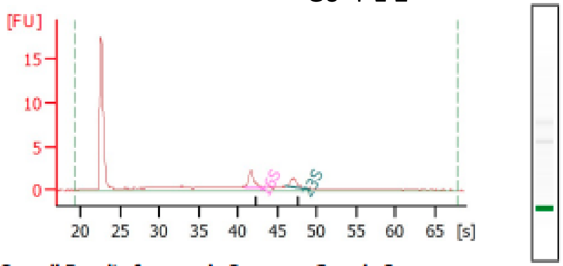

Overall Results for sample 2 : Sample 2

RNA Area:	32.8
RNA Concentration:	32 ng/µl
rRNA Ratio [23s / 16s]:	0.6
RNA Integrity Number (RIN):	7.8 (B.02.09)

Fragment table for sample 2 : Sample 2

Name	Start Time [s]	End Time [s]	Area	% of total Area
16S	40.86	43.67	3.6	11.0
23S	46.08	49.33	2.3	6.9

Go-4-E 3

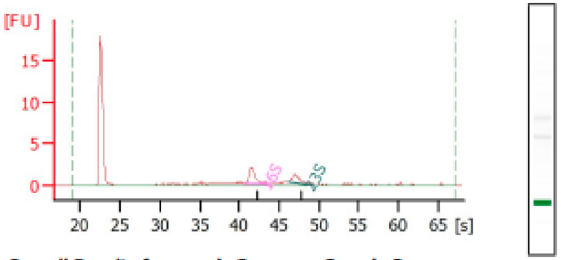

Overall Results for sample 3 : Sample 3

RNA Area:	14.4
RNA Concentration:	14 ng/µl
rRNA Ratio [23s / 16s]:	0.6
RNA Integrity Number (RIN):	8.5 (B.02.09)

Fragment table for sample 3 : Sample 3

Name	Start Time [s]	End Time [s]	Area	% of total Area
16S	40.67	43.78	3.7	25.6
23S	46.12	49.43	2.2	15.3

Dataset 2 - GO-A is synonymous with samples ‘B one’ and GO-E with ‘B two’.

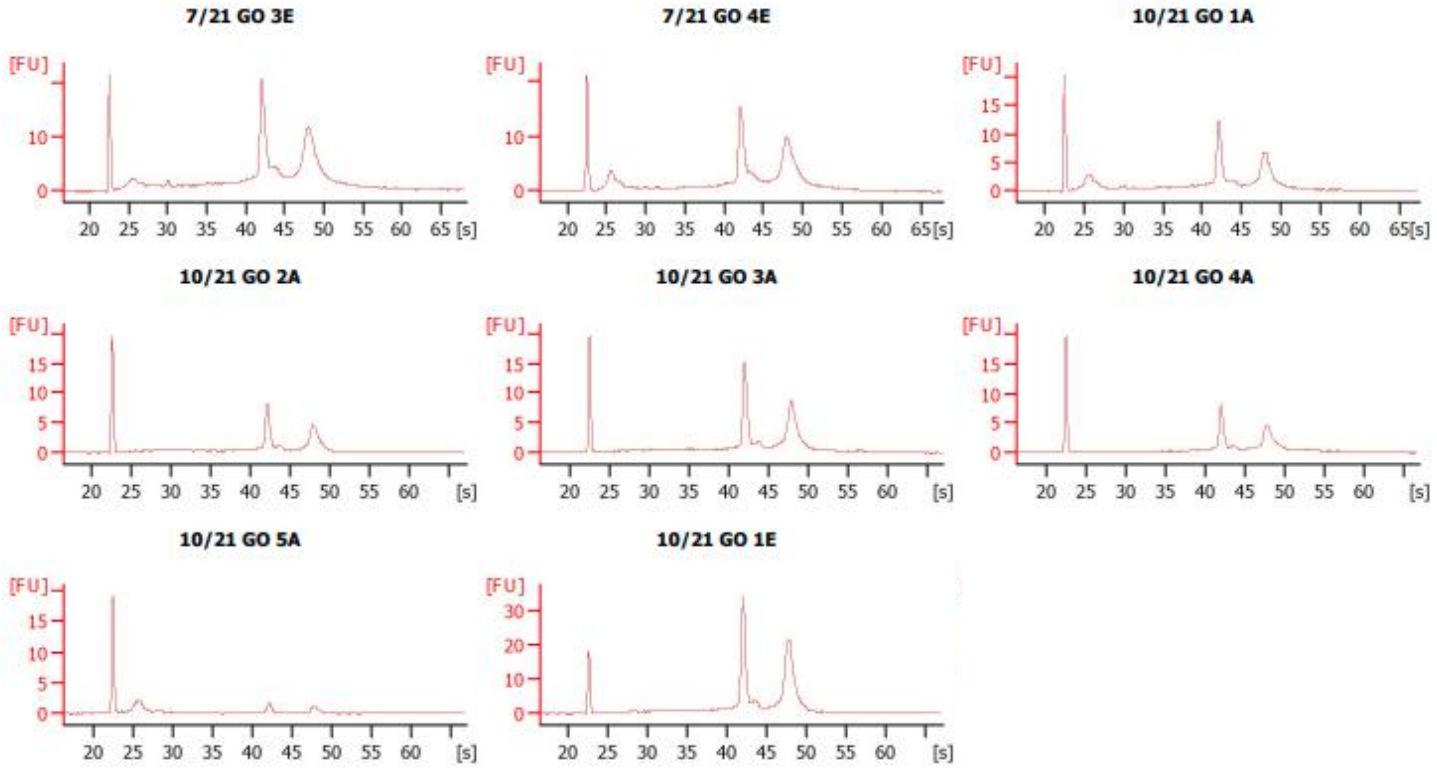

Overall Results for sample 1 : 7/21 GO 3E

RNA Area: 212.7
RNA Concentration: 95 ng/µl
rRNA Ratio [23s / 16s]: 1.0
RNA Integrity Number (RIN): 8.2 (B.02.09)

Fragment table for sample 1 : 7/21 GO 3E

Name	Start Time [s]	End Time [s]	Area	% of total Area
16S	41.15	45.15	37.7	17.7
23S	45.68	53.04	38.5	18.1

Overall Results for sample 2 : 7/21 GO 4E

RNA Area: 161.6
RNA Concentration: 72 ng/µl
rRNA Ratio [23s / 16s]: 1.0
RNA Integrity Number (RIN): 8.7 (B.02.09)

Fragment table for sample 2 : 7/21 GO 4E

Name	Start Time [s]	End Time [s]	Area	% of total Area
16S	41.23	44.81	29.1	18.0
23S	46.06	51.44	30.6	18.9

Overall Results for sample 3 : 10/21 GO 1A

RNA Area: 113.8
RNA Concentration: 51 ng/µl
rRNA Ratio [23s / 16s]: 1.1
RNA Integrity Number (RIN): 8.2 (B.02.09)

Fragment table for sample 3 : 10/21 GO 1A

Name	Start Time [s]	End Time [s]	Area	% of total Area
16S	41.15	43.12	16.5	14.5
23S	45.92	51.12	18.9	16.6

Overall Results for sample 4 : 10/21 GO 2A

RNA Area: 55.7
RNA Concentration: 25 ng/µl
rRNA Ratio [23s / 16s]: 1.0
RNA Integrity Number (RIN): 8.7 (B.02.09)

Fragment table for sample 4 : 10/21 GO 2A

Name	Start Time [s]	End Time [s]	Area	% of total Area
16S	41.35	43.18	11.7	21.1
23S	46.73	51.28	11.3	20.3

Overall Results for sample 5 : 10/21 GO 3A

RNA Area: 94.4
RNA Concentration: 42 ng/µl
rRNA Ratio [23s / 16s]: 0.9
RNA Integrity Number (RIN): 8.9 (B.02.09)

Fragment table for sample 5 : 10/21 GO 3A

... Fragment table for sample 5 : 10/21 GO 3A

Name	Start Time [s]	End Time [s]	Area	% of total Area
16S	41.08	43.09	21.7	23.0
23S	46.72	50.88	19.7	20.9

Overall Results for sample 6 : 10/21 GO 4A

RNA Area: 53.0
RNA Concentration: 24 ng/µl
rRNA Ratio [23s / 16s]: 1.1
RNA Integrity Number (RIN): 9.6 (B.02.09)

Fragment table for sample 6 : 10/21 GO 4A

Name	Start Time [s]	End Time [s]	Area	% of total Area
16S	41.23	42.99	11.2	21.1
23S	46.05	51.40	12.8	24.1

Overall Results for sample 7 : 10/21 GO 5A

RNA Area: 32.2
RNA Concentration: 14 ng/µl
rRNA Ratio [23s / 16s]: 0.9
RNA Integrity Number (RIN): 8.3 (B.02.09)

Fragment table for sample 7 : 10/21 GO 5A

Name	Start Time [s]	End Time [s]	Area	% of total Area
16S	41.27	43.52	3.0	9.3
23S	46.72	49.92	2.7	8.3

Overall Results for sample 8 : 10/21 GO 1E

RNA Area: 186.2
RNA Concentration: 83 ng/µl
rRNA Ratio [23s / 16s]: 1.2
RNA Integrity Number (RIN): 9.3 (B.02.09)

Fragment table for sample 8 : 10/21 GO 1E

Name	Start Time [s]	End Time [s]	Area	% of total Area
16S	40.20	42.99	47.5	25.5
23S	45.82	50.95	58.3	31.3

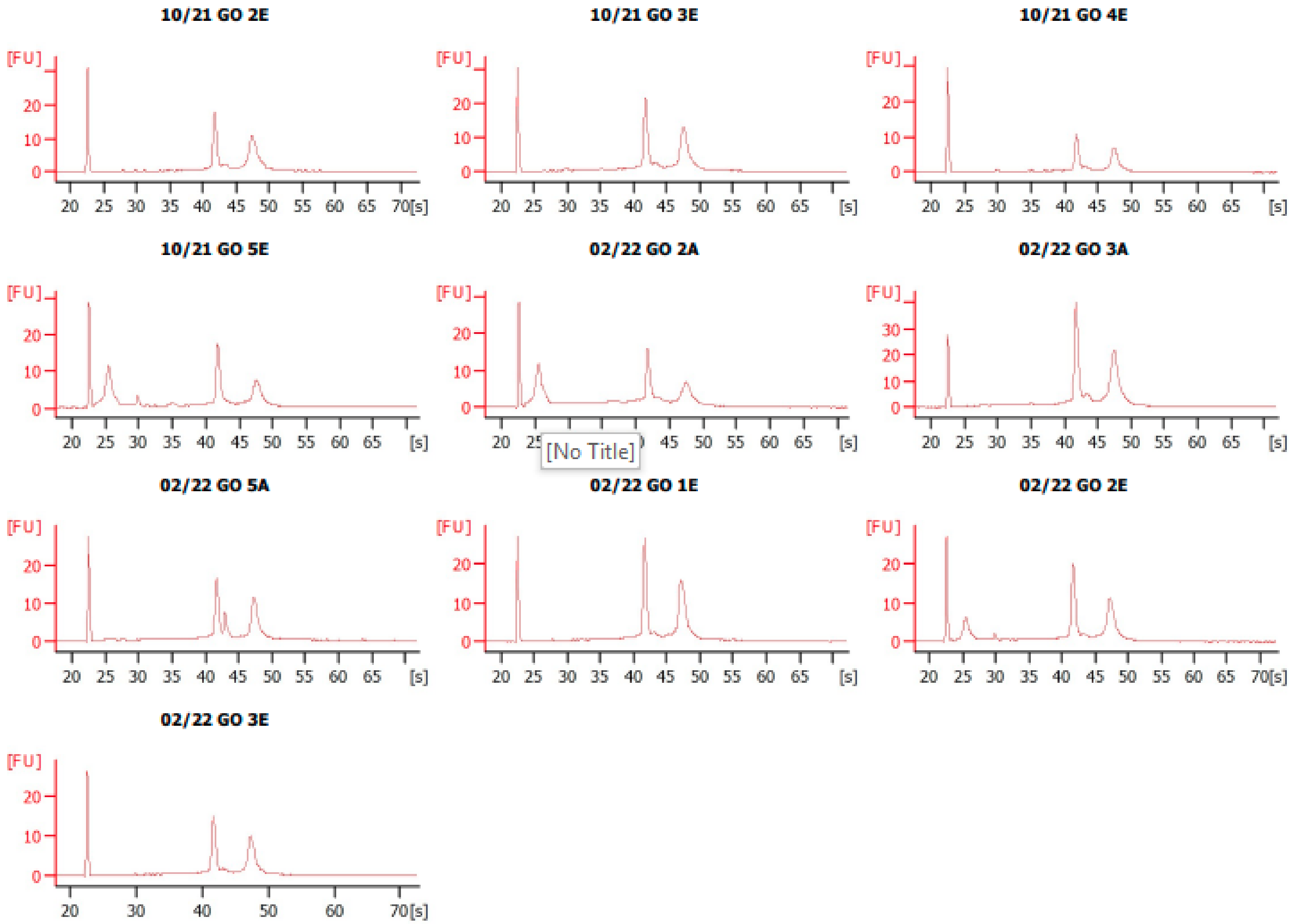
10/21 GO 2E
10/21 GO 3E
10/21 GO 4E
10/21 GO 5E
02/22 GO 2A
02/22 GO 3A
02/22 GO 5A
02/22 GO 1E
02/22 GO 2E
02/22 GO 3E
[No Title]
[FU]
[s]

Overall Results for sample 1 : 10/21 GO 2E

RNA Area: 103.6
RNA Concentration: 26 ng/µl
rRNA Ratio [23s / 16s]: 1.3
RNA Integrity Number (RIN): 9.5 (B.02.09)

Fragment table for sample 1 : 10/21 GO 2E

Name	Start Time [s]	End Time [s]	Area	% of total Area
16S	41.01	42.77	24.5	23.6
23S	45.73	51.12	31.2	30.1

Overall Results for sample 2 : 10/21 GO 3E

RNA Area: 130.9
RNA Concentration: 32 ng/µl
rRNA Ratio [23s / 16s]: 0.9
RNA Integrity Number (RIN): N/A (B.02.09)

Fragment table for sample 2 : 10/21 GO 3E

Name	Start Time [s]	End Time [s]	Area	% of total Area
16S	39.63	44.69	39.7	30.3
23S	45.62	50.68	34.6	26.5

Overall Results for sample 3 : 10/21 GO 4E

RNA Area: 69.0
RNA Concentration: 17 ng/µl
rRNA Ratio [23s / 16s]: 1.1
RNA Integrity Number (RIN): 9.3 (B.02.09)

Fragment table for sample 3 : 10/21 GO 4E

Name	Start Time [s]	End Time [s]	Area	% of total Area
16S	41.04	42.69	15.1	21.9
23S	46.23	50.45	16.3	23.6

Overall Results for sample 4 : 10/21 GO 5E

RNA Area: 161.9
RNA Concentration: 40 ng/µl
rRNA Ratio [23s / 16s]: 0.6
RNA Integrity Number (RIN): 8.8 (B.02.09)

Fragment table for sample 4 : 10/21 GO 5E

Name	Start Time [s]	End Time [s]	Area	% of total Area
16S	40.59	44.49	30.8	19.0
23S	45.92	50.28	19.7	12.2

Overall Results for sample 5 : 02/22 GO 2A

RNA Area: 197.3
RNA Concentration: 49 ng/µl
rRNA Ratio [23s / 16s]: 0.8
RNA Integrity Number (RIN): 7.6 (B.02.09)

Fragment table for sample 5 : 02/22 GO 2A

Name	Start Time [s]	End Time [s]	Area	% of total Area
16S	40.86	42.75	22.1	11.2
23S	45.92	50.73	18.8	9.5

Overall Results for sample 6 : 02/22 GO 3A

RNA Area: 236.4
RNA Concentration: 59 ng/µl
rRNA Ratio [23s / 16s]: 1.1
RNA Integrity Number (RIN): 9.3 (B.02.09)

Fragment table for sample 6 : 02/22 GO 3A

Name	Start Time [s]	End Time [s]	Area	% of total Area
16S	39.86	42.74	58.0	24.5
23S	45.00	50.80	66.4	28.1

Overall Results for sample 7 : 02/22 GO 5A

RNA Area: 105.7
RNA Concentration: 26 ng/µl
rRNA Ratio [23s / 16s]: 1.2
RNA Integrity Number (RIN): 9.1 (B.02.09)

Fragment table for sample 7 : 02/22 GO 5A

Name	Start Time [s]	End Time [s]	Area	% of total Area
16S	40.63	42.53	21.8	20.7
23S	45.72	50.80	26.8	25.4

Overall Results for sample 8 : 02/22 GO 1E

RNA Area: 139.7
RNA Concentration: 35 ng/µl
rRNA Ratio [23s / 16s]: 1.2
RNA Integrity Number (RIN): 9.5 (B.02.09)

Fragment table for sample 8 : 02/22 GO 1E

Name	Start Time [s]	End Time [s]	Area	% of total Area
16S	40.20	42.68	37.0	26.5
23S	44.38	50.68	44.0	31.5

Overall Results for sample 9 : 02/22 GO 2E

RNA Area: 137.4
RNA Concentration: 34 ng/µl
rRNA Ratio [23s / 16s]: 1.1
RNA Integrity Number (RIN): 9 (B.02.09)

Fragment table for sample 9 : 02/22 GO 2E

Name	Start Time [s]	End Time [s]	Area	% of total Area
16S	40.85	42.77	29.3	21.3
23S	44.64	50.29	32.8	23.9

Overall Results for sample 10 : 02/22 GO 3E

RNA Area: 90.4
RNA Concentration: 22 ng/µl
rRNA Ratio [23s / 16s]: 1.1
RNA Integrity Number (RIN): 9.2 (B.02.09)

... Fragment table for sample 10 : 02/22 GO 3E

Name	Start Time [s]	End Time [s]	Area	% of total Area
16S	40.69	42.77	22.0	24.3
23S	45.68	50.72	25.1	27.7

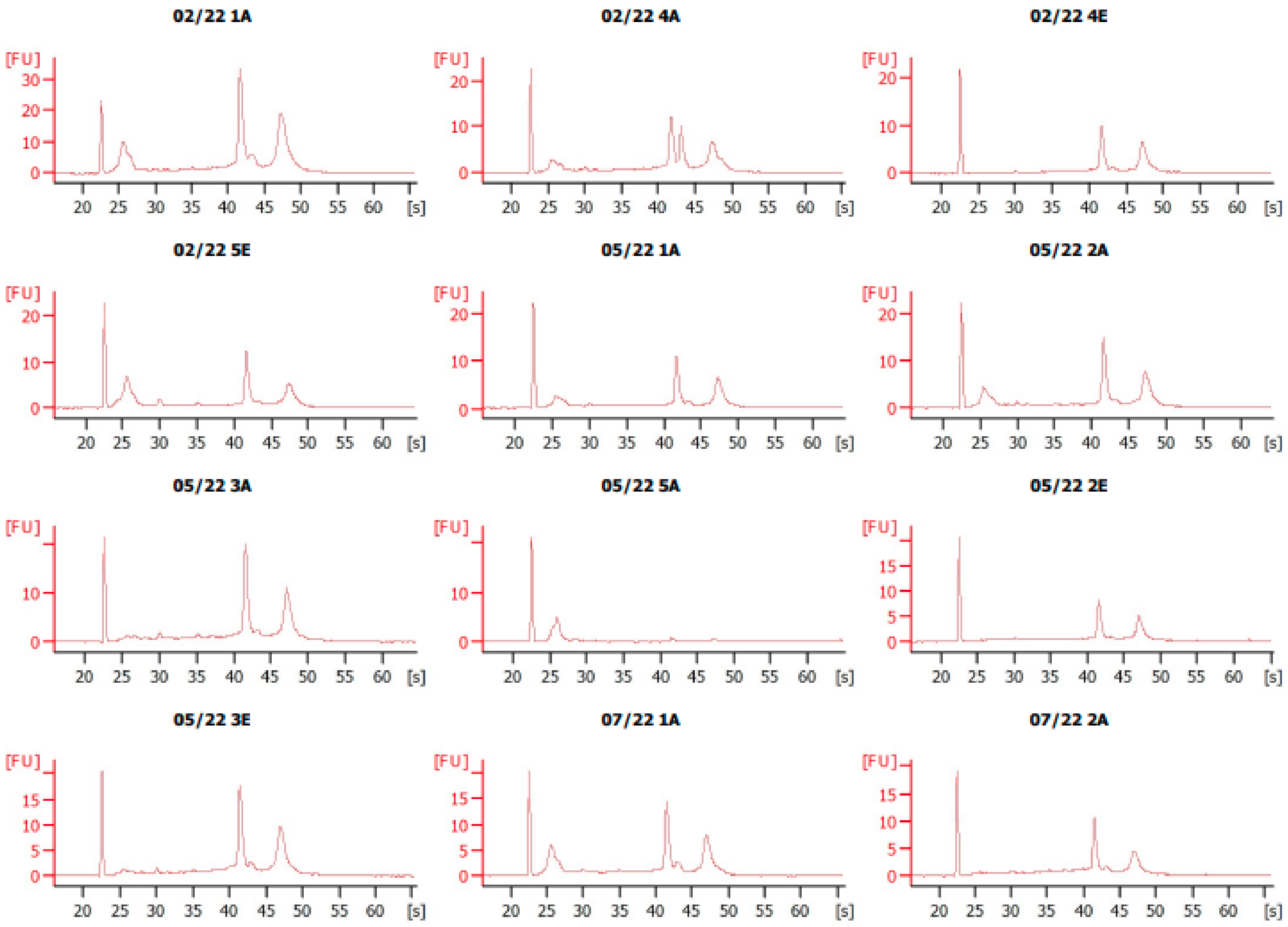
02/22 1A
02/22 4A
02/22 4E
02/22 5E
05/22 1A
05/22 2A
05/22 3A
05/22 5A
05/22 2E
05/22 3E
07/22 1A
07/22 2A
[FU]
[s]
20 25 30 35 40 45 50 55 60

Overall Results for sample 1 : 02/22 1A

RNA Area:	286.5
RNA Concentration:	110 ng/µl
rRNA Ratio [23s / 16s]:	1.3
RNA Integrity Number (RIN):	8.5 (B.02.09)

Fragment table for sample 1 : 02/22 1A

Name	Start Time [s]	End Time [s]	Area	% of total Area
16S	40.40	42.60	45.5	15.9
23S	44.72	50.78	59.6	20.8

Overall Results for sample 2 : 02/22 4A

RNA Area:	127.6
RNA Concentration:	49 ng/µl
rRNA Ratio [23s / 16s]:	1.3
RNA Integrity Number (RIN):	8.1 (B.02.09)

Fragment table for sample 2 : 02/22 4A

Name	Start Time [s]	End Time [s]	Area	% of total Area
16S	40.99	42.62	15.3	12.0
23S	45.51	50.55	19.9	15.6

Overall Results for sample 3 : 02/22 4E

RNA Area:	54.1
RNA Concentration:	21 ng/µl
rRNA Ratio [23s / 16s]:	1.0
RNA Integrity Number (RIN):	8.9 (B.02.09)

Fragment table for sample 3 : 02/22 4E

Name	Start Time [s]	End Time [s]	Area	% of total Area
16S	40.74	42.69	13.5	24.9
23S	46.11	50.42	14.0	25.9

Overall Results for sample 4 : 02/22 5E

RNA Area:	117.9
RNA Concentration:	45 ng/µl
rRNA Ratio [23s / 16s]:	0.8
RNA Integrity Number (RIN):	8.1 (B.02.09)

Fragment table for sample 4 : 02/22 5E

Name	Start Time [s]	End Time [s]	Area	% of total Area
16S	40.81	42.75	16.7	14.2
23S	45.97	50.11	13.0	11.1

Overall Results for sample 5 : 05/22 1A

RNA Area:	89.2
RNA Concentration:	34 ng/µl
rRNA Ratio [23s / 16s]:	0.9
RNA Integrity Number (RIN):	8.5 (B.02.09)

Fragment table for sample 5 : 05/22 1A

Name	Start Time [s]	End Time [s]	Area	% of total Area
16S	40.97	42.80	15.1	16.9

... Fragment table for sample 5 : 05/22 1A

Name	Start Time [s]	End Time [s]	Area	% of total Area
23S	46.15	50.50	14.4	16.1

Overall Results for sample 6 : 05/22 2A

RNA Area:	116.6
RNA Concentration:	45 ng/µl
rRNA Ratio [23s / 16s]:	1.0
RNA Integrity Number (RIN):	8.4 (B.02.09)

Fragment table for sample 6 : 05/22 2A

Name	Start Time [s]	End Time [s]	Area	% of total Area
16S	40.82	42.70	20.3	17.4
23S	45.55	50.69	19.6	16.8

Overall Results for sample 7 : 05/22 3A

RNA Area:	129.3
RNA Concentration:	50 ng/µl
rRNA Ratio [23s / 16s]:	0.8
RNA Integrity Number (RIN):	8.5 (B.02.09)

Fragment table for sample 7 : 05/22 3A

Name	Start Time [s]	End Time [s]	Area	% of total Area
16S	39.33	42.74	30.0	23.2
23S	45.46	50.35	25.3	19.6

Overall Results for sample 8 : 05/22 5A

RNA Area:	31.3
RNA Concentration:	12 ng/µl
rRNA Ratio [23s / 16s]:	0.0
RNA Integrity Number (RIN):	2.5 (B.02.09)

Fragment table for sample 8 : 05/22 5A

Name	Start Time [s]	End Time [s]	Area	% of total Area
16S	40.92	42.82	1.3	4.2

Overall Results for sample 9 : 05/22 2E

RNA Area:	54.4
RNA Concentration:	21 ng/µl
rRNA Ratio [23s / 16s]:	0.9
RNA Integrity Number (RIN):	8.3 (B.02.09)

Fragment table for sample 9 : 05/22 2E

Name	Start Time [s]	End Time [s]	Area	% of total Area
16S	40.90	42.76	10.8	19.8
23S	46.12	49.57	9.3	17.1

Overall Results for sample 10 : 05/22 3E

RNA Area:	117.1
RNA Concentration:	45 ng/µl
rRNA Ratio [23s / 16s]:	1.2
RNA Integrity Number (RIN):	8.3 (B.02.09)

Overall Results for sample 11 : 07/22 1A

RNA Area:	128.4
RNA Concentration:	49 ng/µl
rRNA Ratio [23s / 16s]:	1.1
RNA Integrity Number (RIN):	8.3 (B.02.09)

Fragment table for sample 11 : 07/22 1A

Name	Start Time [s]	End Time [s]	Area	% of t[illegible]
16S	40.25	42.41	19.6	15.2
23S	45.28	50.37	20.7	16.1

Overall Results for sample 12 : 07/22 2A

RNA Area:	79.1
RNA Concentration:	30 ng/µl
rRNA Ratio [23s / 16s]:	0.8
RNA Integrity Number (RIN):	7.7 (B.02.09)

Fragment table for sample 12 : 07/22 2A

Name	Start Time [s]	End Time [s]	Area	% of t[illegible]
16S	40.71	42.55	13.1	16.6
23S	45.71	50.09	10.5	13.2

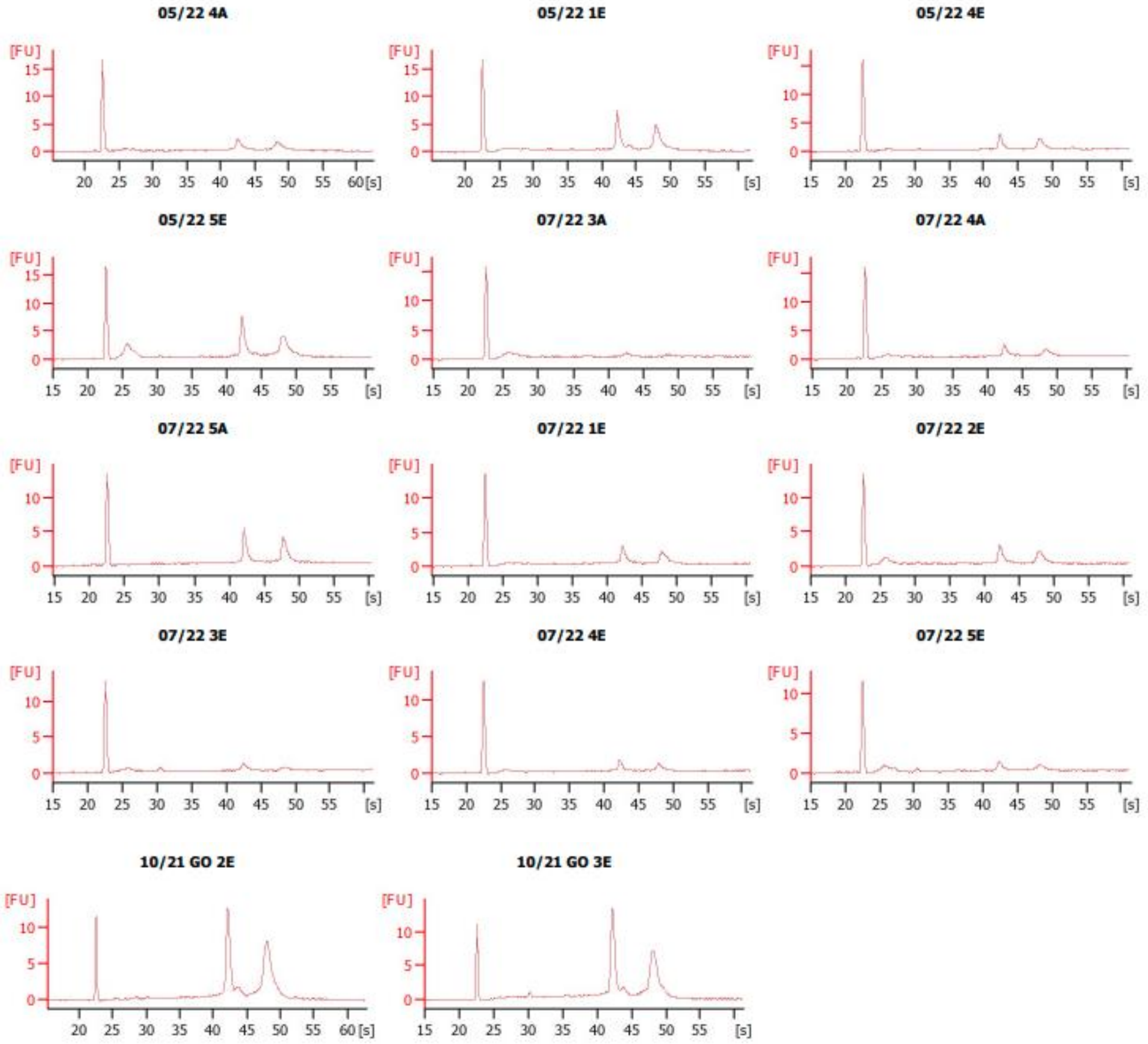
05/22 4A
05/22 1E
05/22 4E
05/22 5E
07/22 3A
07/22 4A
07/22 5A
07/22 1E
07/22 2E
07/22 3E
07/22 4E
07/22 5E
10/21 GO 2E
10/21 GO 3E
[FU]
[s]

Overall Results for sample 1 : 05/22 4A

RNA Area: 36.7
RNA Concentration: 31 ng/µl
rRNA Ratio [23s / 16s]: 1.0
RNA Integrity Number (RIN): 7.9 (B.02.09)

Fragment table for sample 1 : 05/22 4A

Name	Start Time [s]	End Time [s]	Area	% of total Area
16S	41.70	43.98	3.6	9.8
23S	47.36	50.70	3.6	9.8

Overall Results for sample 2 : 05/22 1E

RNA Area: 50.1
RNA Concentration: 42 ng/µl
rRNA Ratio [23s / 16s]: 1.1
RNA Integrity Number (RIN): 8.4 (B.02.09)

Fragment table for sample 2 : 05/22 1E

Name	Start Time [s]	End Time [s]	Area	% of total Area
16S	41.60	43.54	9.6	19.1
23S	46.72	51.54	10.3	20.5

Overall Results for sample 3 : 05/22 4E

RNA Area: 19.0
RNA Concentration: 16 ng/µl
rRNA Ratio [23s / 16s]: 0.9
RNA Integrity Number (RIN): 8.8 (B.02.09)

Fragment table for sample 3 : 05/22 4E

Name	Start Time [s]	End Time [s]	Area	% of total Area
16S	41.76	43.60	3.9	20.3
23S	47.45	50.43	3.5	18.1

Overall Results for sample 4 : 05/22 5E

RNA Area: 63.9
RNA Concentration: 54 ng/µl
rRNA Ratio [23s / 16s]: 0.8
RNA Integrity Number (RIN): 8.9 (B.02.09)

Fragment table for sample 4 : 05/22 5E

Name	Start Time [s]	End Time [s]	Area	% of total Area
16S	41.43	43.65	11.0	17.2
23S	47.09	51.01	9.2	14.4

Overall Results for sample 5 : 07/22 3A

RNA Area: 28.9
RNA Concentration: 24 ng/µl
rRNA Ratio [23s / 16s]: 0.0
RNA Integrity Number (RIN): 1 (B.02.09)

Overall Results for sample 6 : 07/22 4A

RNA Area: 21.9
RNA Concentration: 18 ng/µl

Fragment table for sample 6 : 07/22 4A

Name	Start Time [s]	End Time [s]	Area	% of total Area
16S	41.76	43.88	3.3	15.2
23S	47.72	50.48	2.1	9.3

Overall Results for sample 7 : 07/22 5A

RNA Area: 35.0
RNA Concentration: 30 ng/µl
rRNA Ratio [23s / 16s]: 0.9
RNA Integrity Number (RIN): 8.8 (B.02.09)

Fragment table for sample 7 : 07/22 5A

Name	Start Time [s]	End Time [s]	Area	% of total Area
16S	41.37	44.44	7.5	21.5
23S	46.90	50.10	6.7	19.0

Overall Results for sample 8 : 07/22 1E

RNA Area: 25.7
RNA Concentration: 22 ng/µl
rRNA Ratio [23s / 16s]: 1.0
RNA Integrity Number (RIN): 8.3 (B.02.09)

Fragment table for sample 8 : 07/22 1E

Name	Start Time [s]	End Time [s]	Area	% of total Area
16S	41.66	43.78	4.1	16.1
23S	47.22	50.69	4.1	16.0

Overall Results for sample 9 : 07/22 2E

RNA Area: 30.2
RNA Concentration: 25 ng/µl
rRNA Ratio [23s / 16s]: 0.9
RNA Integrity Number (RIN): 8.4 (B.02.09)

Fragment table for sample 9 : 07/22 2E

Name	Start Time [s]	End Time [s]	Area	% of total Area
16S	41.42	43.60	4.7	15.4
23S	47.01	50.41	4.4	14.5

Overall Results for sample 10 : 07/22 3E

RNA Area: 15.6
RNA Concentration: 13 ng/µl
rRNA Ratio [23s / 16s]: 0.0
RNA Integrity Number (RIN): 7.2 (B.02.09)

Fragment table for sample 10 : 07/22 3E

Name	Start Time [s]	End Time [s]	Area	% of total Area
16S	41.72	44.04	2.1	13.4

Overall Results for sample 11 : 07/22 4E

RNA Area: 11.9
RNA Concentration: 10 ng/µl
rRNA Ratio [23s / 16s]: 0.8
RNA Integrity Number (RIN): 8.6 (B.02.09)

Fragment table for sample 11 : 07/22 4E

Name	Start Time [s]	End Time [s]	Area
16S	41.45	44.56	2.6
23S	47.15	50.35	2.2

Overall Results for sample 12 : 07/22 5E

RNA Area: 20.1
RNA Concentration: 17 ng/µl
rRNA Ratio [23s / 16s]: 0.0
RNA Integrity Number (RIN): 7.1 (B.02.09)

Fragment table for sample 12 : 07/22 5E

Name	Start Time [s]	End Time [s]	Area
16S	41.58	44.12	2.6

Overall Results for sample 1 : 10/21 GO 2E

RNA Area: 72.6
RNA Concentration: 35 ng/µl
rRNA Ratio [23s / 16s]: 1.3
RNA Integrity Number (RIN): 9.1 (B.02.09)

Fragment table for sample 1 : 10/21 GO 2E

Name	Start Time [s]	End Time [s]	Area	[illegible]
16S	41.32	43.16	17.2	[illegible]
23S	46.25	50.69	22.6	[illegible]

Overall Results for sample 2 : 10/21 GO 3E

RNA Area: 88.1
RNA Concentration: 42 ng/µl
rRNA Ratio [23s / 16s]: 1.1
RNA Integrity Number (RIN): 8.5 (B.02.09)

Fragment table for sample 2 : 10/21 GO 3E

Name	Start Time [s]	End Time [s]	Area	[illegible]
16S	41.46	43.38	18.6	[illegible]
23S	46.44	51.60	19.8	[illegible]

Dataset 4 – NAE$_{std}$ *S. solfataricus* pure culture.

Several extractions were used, all had comparable quality.

S. Solfataricus 1

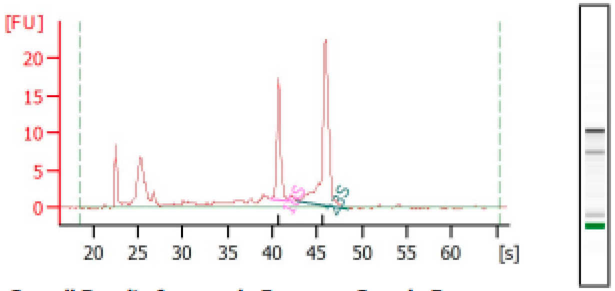

Overall Results for sample 5 : **Sample 5**

RNA Area:	135.9
RNA Concentration:	43 ng/µl
rRNA Ratio [23s / 16s]:	2.2
RNA Integrity Number (RIN):	8.7 (B.02.09)

Fragment table for sample 5 : **Sample 5**

Name	Start Time [s]	End Time [s]	Area	% of total Area
16S	39.69	41.73	18.8	13.9
23S	42.63	48.46	41.1	30.2

S. Solfataricus 2

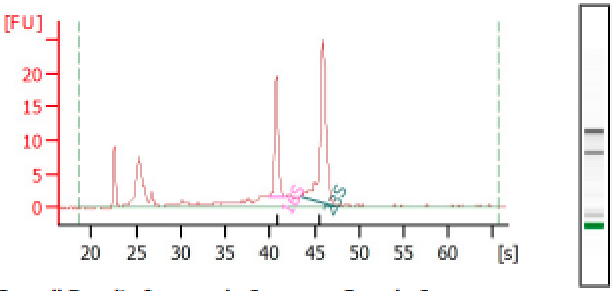

Overall Results for sample 6 : **Sample 6**

RNA Area:	146.8
RNA Concentration:	47 ng/µl
rRNA Ratio [23s / 16s]:	2.2
RNA Integrity Number (RIN):	8.7 (B.02.09)

Fragment table for sample 6 : **Sample 6**

Name	Start Time [s]	End Time [s]	Area	% of total Area
16S	40.01	41.67	19.9	13.6
23S	43.43	47.57	43.7	29.8

S solf 3

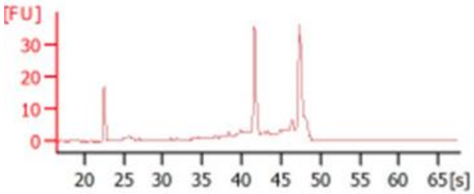

S solf 1

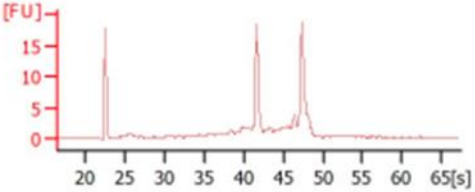

S solf 2

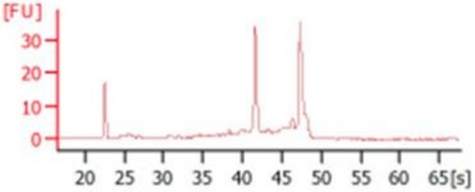

Overall Results for sample 10 : S solf 1

RNA Area:	90.1
RNA Concentration:	40 ng/µl
rRNA Ratio [23s / 16s]:	1.2
RNA Integrity Number (RIN):	8.3 (B.02.09)

... Fragment table for sample 10 : **S solf 1**

Name	Start Time [s]	End Time [s]	Area	% of total Area
16S	40.96	42.40	16.7	18.5
23S	46.78	48.99	20.1	22.3

Overall Results for sample 11 : S solf 2

RNA Area:	161.5
RNA Concentration:	72 ng/µl
rRNA Ratio [23s / 16s]:	1.2
RNA Integrity Number (RIN):	8.2 (B.02.09)

Fragment table for sample 11 : **S solf 2**

Name	Start Time [s]	End Time [s]	Area	% of total Area
16S	40.80	42.49	31.6	19.6
23S	46.69	48.91	38.8	24.0

Overall Results for sample 12 : S solf 3

RNA Area:	164.6
RNA Concentration:	73 ng/µl
rRNA Ratio [23s / 16s]:	1.2
RNA Integrity Number (RIN):	8.3 (B.02.09)

Fragment table for sample 12 : **S solf 3**

Name	Start Time [s]	End Time [s]	Area	% of total Area
16S	40.80	42.49	31.4	19.1
23S	46.79	48.96	38.8	23.6
