# Supplementary material for: Quantifying Soil Microbiome Abundance by Metatranscriptomics and Complementary Molecular Techniques—Cross‐Validation and Perspectives
Source: Mol Ecol Resour. 2025 Jun 3;25(7):e14130. doi: 10.1111/1755-0998.14130 (PMC12415835; doi:10.1111/1755-0998.14130)
Supplement: Supplementary file 6 — Data S6. Context data relevant for the estimated biomasses. [file MEN-25-e14130-s001.pdf]

Supplement S6
Context data relevant for the estimated biomasses

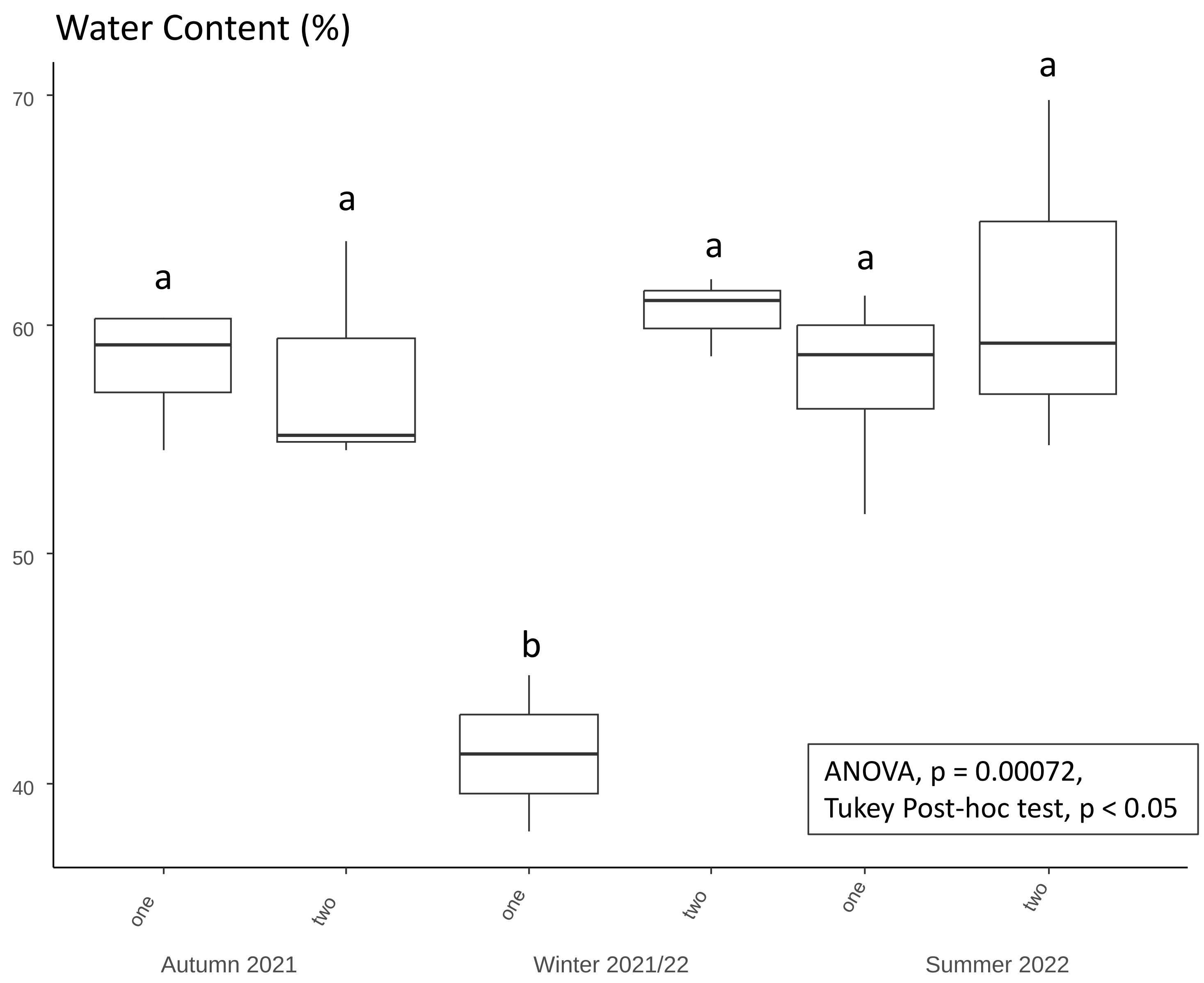
Water Content (%)
70
60
50
40
a
a
b
a
a
a
one
two
one
two
one
two
Autumn 2021
Winter 2021/22
Summer 2022
ANOVA, p = 0.00072,
Tukey Post-hoc test, p < 0.05

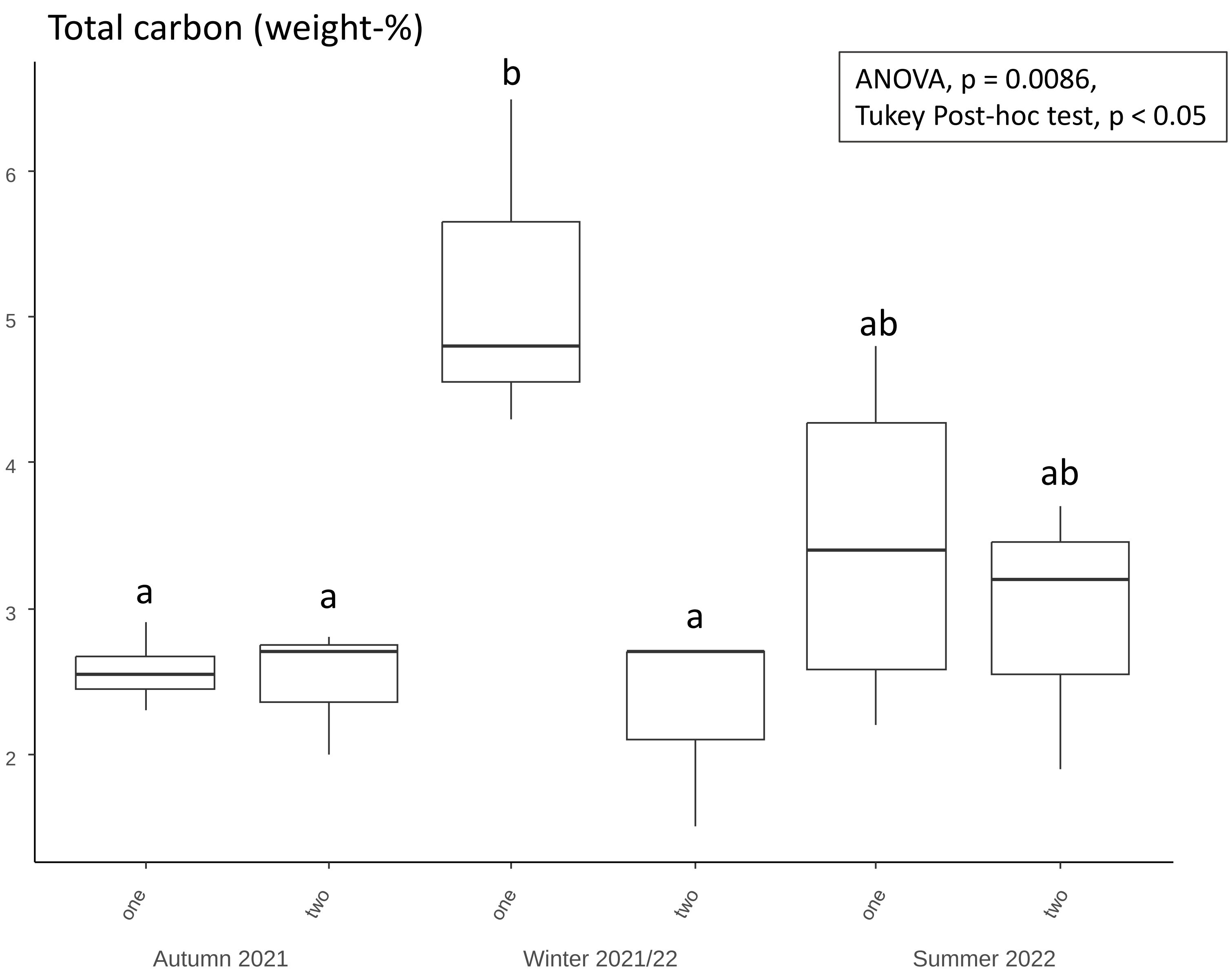
Total carbon (weight-%)
ANOVA, p = 0.0086,
Tukey Post-hoc test, p < 0.05
b
ab
ab
a
a
a
6
5
4
3
2
one
two
one
two
one
two
Autumn 2021
Winter 2021/22
Summer 2022

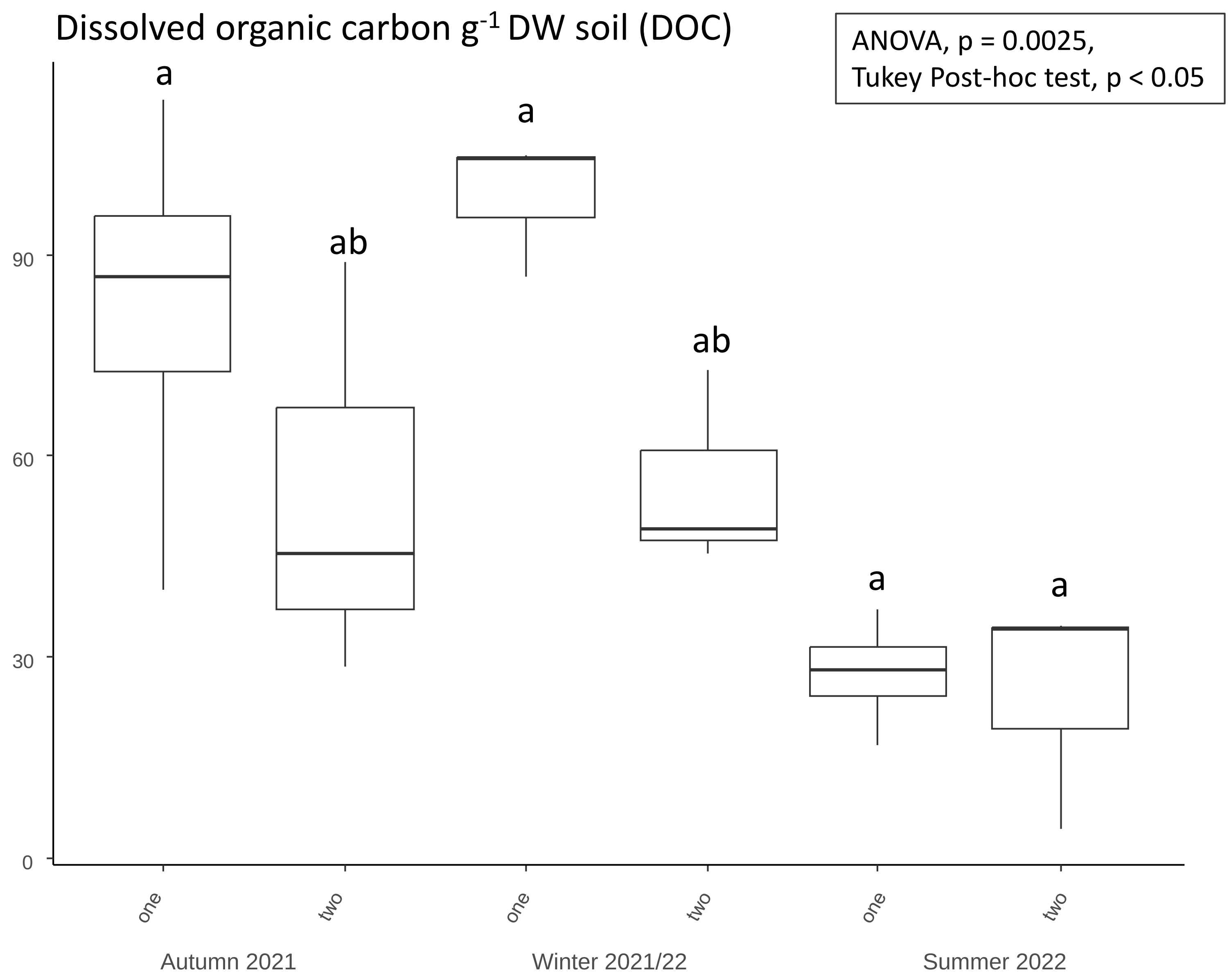
Dissolved organic carbon g^{-1} DW soil (DOC)
ANOVA, p = 0.0025,
Tukey Post-hoc test, p < 0.05
a
ab
a
ab
a
a
90
60
30
0
one
two
one
two
one
two
Autumn 2021
Winter 2021/22
Summer 2022

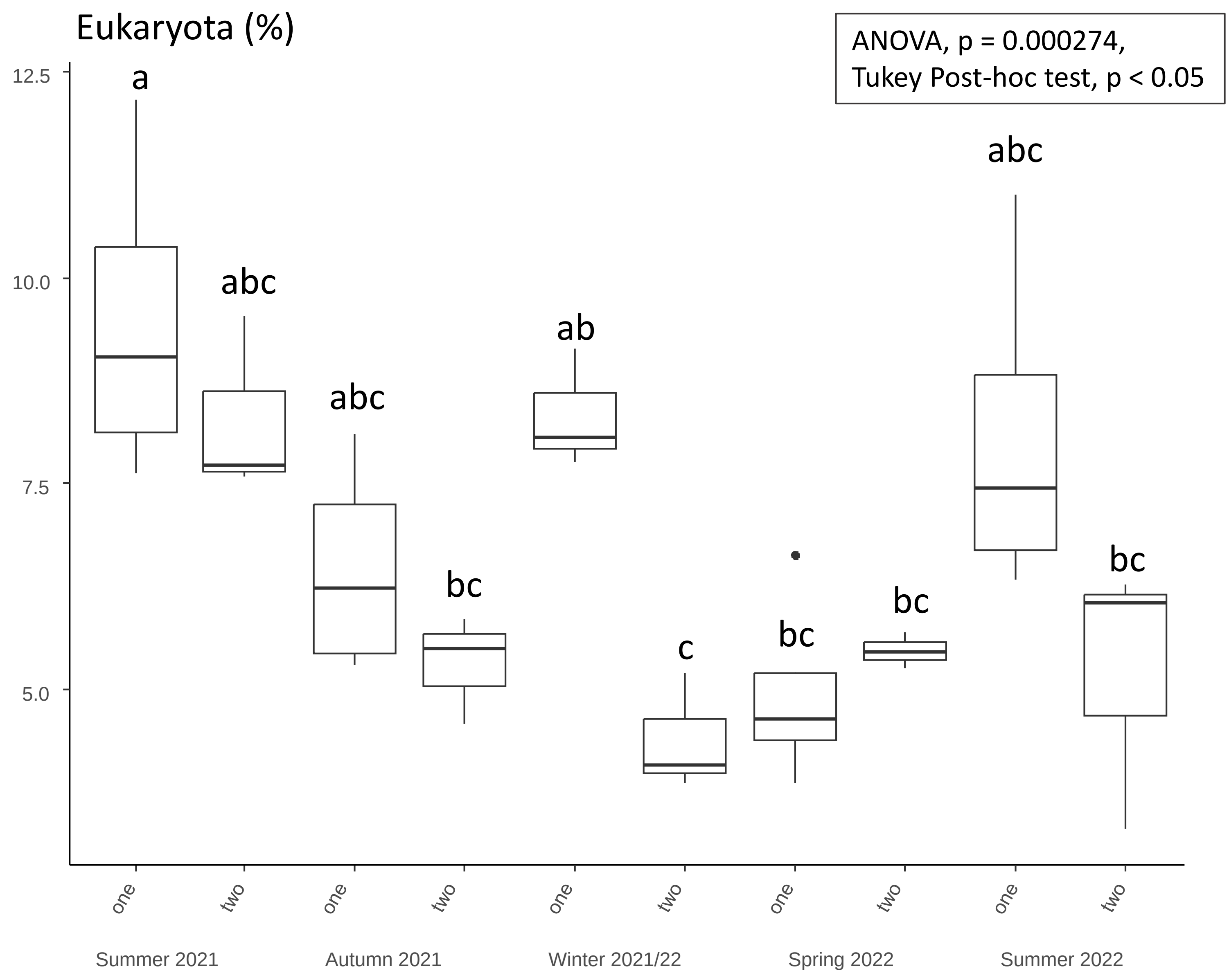
Eukaryota (%)
ANOVA, p = 0.000274,
Tukey Post-hoc test, p < 0.05
12.5
10.0
7.5
5.0
a
abc
abc
bc
ab
c
bc
bc
abc
bc
one
two
one
two
one
two
one
two
one
two
Summer 2021
Autumn 2021
Winter 2021/22
Spring 2022
Summer 2022
